# Nucleophilic Reactivity at a =CH Arm of a Lutidine-Based CNC/Rh System: Unusual Alkyne and CO_2_ Activation

**DOI:** 10.1021/acs.inorgchem.2c00617

**Published:** 2022-04-27

**Authors:** Pablo Hermosilla, Pilar García-Orduña, Pablo J. Sanz Miguel, Víctor Polo, Miguel A. Casado

**Affiliations:** † Departamento de Química Inorgánica, Instituto de Síntesis Química y Catálisis Homogénea (ISQCH), Universidad de Zaragoza-CSIC, 50009 Zaragoza, Spain; ‡ Departamento de Química Física and Instituto de Biocomputación y Física de los Sistemas Complejos (BIFI), Universidad de Zaragoza, 50009 Zaragoza, Spain

## Abstract

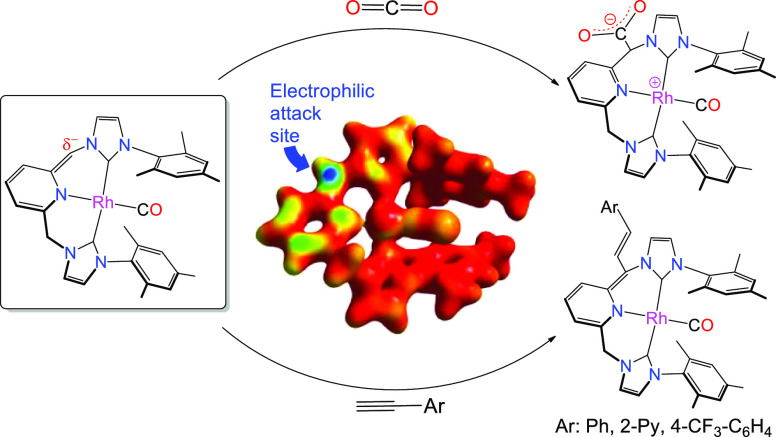

Reaction of an amido pincer complex [(CNC)*Rh(CO)] (**1**) (CNC* is the deprotonated form of CNC) with carbon dioxide gave a neutral complex [(CNC-CO_2_)^Mes^*Rh(CO)] (**2**), which is the result of a C–C bond-forming reaction between the deprotonated arm of the CNC* ligand and CO_2_. The molecular structure of **2** showed a zwitterionic complex, where the CO_2_ moiety is covalently connected to the former =CH arm of the CNC* pincer ligand. The unusual structure of **1** allowed us to explore the reactivity of the CO_2_ moiety with selected primary amines RNH_2_ (benzylamine and ammonia), which afforded cationic complexes [(CNC)^Mes^Rh(CO)][HRNC(*O*)O] (R = Bz (**3**), H (**4**)). Compounds **3** and **4** are the result of a C–N coupling between the incoming amine and the CO_2_ fragment covalently connected to the pincer ligand in **2**, a process that involves protonation of the “CH–CO_2_” fragment in **2** from the respective amines. Once revealed the nucleophilic character of the =CH fragment in **1**, we explored its reactivity with alkynes, a study that enlightened a novel reactivity trend in alkyne activation. Reaction of **1** with terminal alkynes RC≡CH (R = Ph, 2-py, 4-C_6_H_4_-CF_3_) yielded neutral complexes [(CNC-CH=CHR)^Mes^*Rh(CO)] (R = Ph (**5**), 2-py (**6**), 4-C_6_H_4_-CF_3_ (**7**)) in good yields. Deuterium labeling experiments with PhC≡CD confirmed that complex **5** is the product of a formal insertion of the alkyne into the C(sp^2^)–H bond of the deprotonated arm in **1**. This structural proposal was further confirmed by the X-ray molecular structure of phenyl complex **5**, which showed the alkyne covalently linked to the pincer ligand. Besides, this novel transformation was analyzed by DFT methods and showed a metal–ligand cooperative mechanism, based on the initial electrophilic attack of the alkyne to the =CH arm of the CNC^Mes^* ligand (making a new C–C bond) followed by the action of a protic base (HN(SiMe_3_)_2_), which is able to perform a proton rearrangement that leads to the final product **5**.

## Abstract 2

This study explores the reactivity of a carbonyl rhodium(I) complex stabilized with a deprotonated CNC^Mes^* pincer ligand with CO_2_ and terminal alkynes.

Pincer ligands in combination with late transition metals are one of the most utilized strategies to access highly active systems both in catalysis^1^ and bond activation.^2^ To this date, there have been an important number of examples of catalytic processes proceeding through metal–ligand cooperation.^3^ There are relatively new catalytic processes based on pyridine-based ligands, which have the potential of undergoing an aromatization–dearomatization sequence that established the foundation of modern catalysis.^4^ A relevant aspect within the context of homogeneous catalysis is concerned with the stability of the auxiliary ligands under the catalytic conditions used, which may affect the outcome or even deactivate some desired catalytic transformations.^5^

Specifically, in some deprotonated lutidine-based transition metal pincer complexes, the =CH arm connected to the pyridinic central core is prone to undergo C–C bond-forming reactions. For instance, a PNP*-based Cu complex (PNP* = deprotonated form of PNP; see Scheme 1 for identity of its structure) becomes methylated with MeOTf;^6^ PNP* Ru^7^ and Mn^8^ complexes undergo reversible C–C coupling with alcohols in which the aldehydes generated upon alcohol dehydrogenation are trapped as the corresponding alkoxo complexes. PNP* Re^9^ and PNN* and CNC Ru^10^ complexes readily activate nitriles and different carbonyl compounds^11^ by reversible C–C bond formation processes. Interestingly, Milstein et al. have recently reported the Michael addition reactions of nonactivated aliphatic nitriles to α,β-unsaturated carbonyl compounds catalyzed by PNP* Mn complexes.^12^ In the same line, de Vries et al. have reported a PNN* Ru complex that catalyzes the oxa Michael addition to unsaturated nitriles.^13^ DFT studies strongly indicate that in these catalytic transformations, the cooperation between the metal and the corresponding dearomatized ligand leads to the generation of metalated nitrile nucleophile species, where the release of the respective organic product involves a C–C bond breaking process. Along this line, the reactivity and catalysis of transition metal, pincer-based complexes with carbon dioxide is a hot topic.^14^

**Scheme 1:**
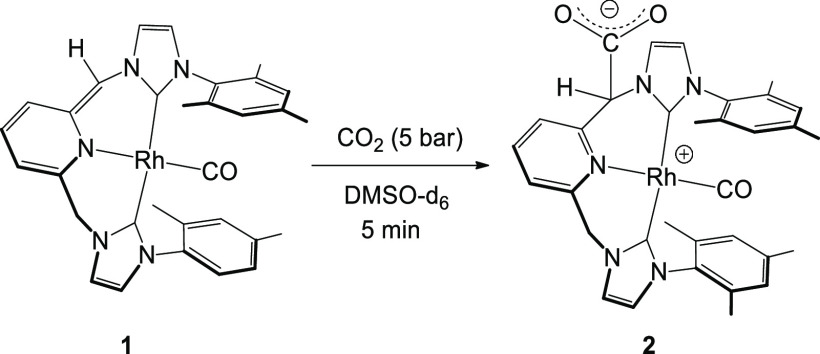
Synthesis of Complex **2**

In this contribution, we deal with a deprotonated CNC-based pincer in combination with an “Rh(CO)” organometallic fragment featuring a “=CH” arm, namely, [(CNC)*Rh(CO)],^15^ that displays an interesting nucleophilic reactivity with some substrates such as carbon dioxide and terminal alkynes, illustrating novel reactivity patterns that have been studied by a combination of spectroscopic techniques and DFT theoretical methods.

## Results and Discussion

In general, the unsaturated **=**CHN arm in deprotonated, neutral complexes stabilized with lutidine-based pincer ligands has a nucleophilic character, while the metal may behave as an electrophilic center in possible cooperative processes that do not involve a change in the oxidation state of the metal.^16^ We recently reported the synthesis of a bis(carbene) lutidine-based rhodium complex [(CNC)^Mes^*Rh(CO)] (**1**),^15^ and in this contribution, we explore the reactivity of the **=**CH arm in **1** with a variety of electrophiles, which include CO_2_ and some terminal alkynes.

Exposure of a solution of **1** in DMSO-d_6_ under an atmosphere of carbon dioxide for 5 min afforded new species, which was further characterized as zwitterionic complex [(CNC-CO_2_)^Mes^*Rh(CO)] (**2**) both in solution and in the solid state (Scheme 1). Complex **2** was not soluble in most of the organic solvents tested; however, it was soluble enough in DMSO to run a complete series of nuclear magnetic resonance (NMR) experiments in solution.

**Figure 1:**
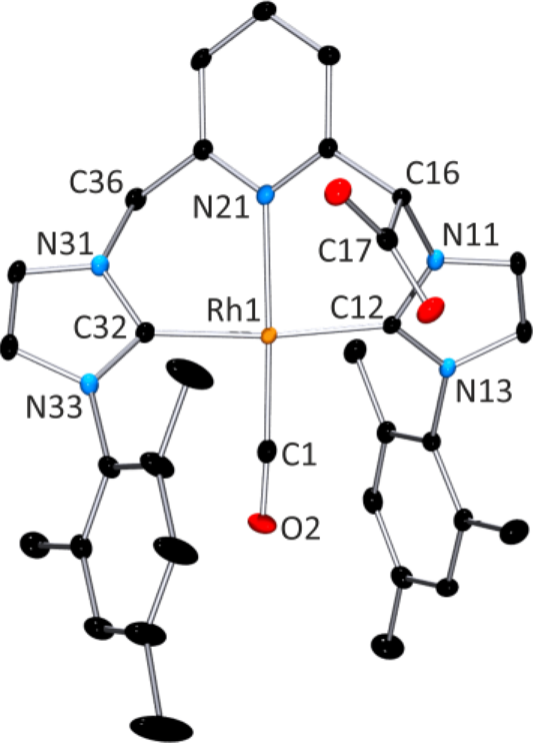
Molecular structure of compound **2**, [(CNC-CO_2_)^Mes*^Rh(CO)]·3DMSO. Hydrogen atoms and DMSO molecules have been omitted for clarity. Selected distances: Rh1–C1, 1.809(2) Å; Rh1–C12, 2.034(2) Å; Rh1–C32, 2.025(2) Å; Rh1–N21, 2.1579(18) Å; and C1–O2 1.152(3) Å. Selected angles C1–Rh1–C12, 94.61(10)°; C1–Rh1–N21, 175.74(10)°; C1–Rh1–C32, 91.75(10)°; C12–Rh1–N21 87.26(8)°; C12–Rh1–C32, 173.31(9)°; N21–Rh1–C32, 86.54(8)°; Rh1–C1–O2, 176.6(2)°.

The ^1^H NMR image of **2** in DMSO-d_6_ showed the pyridinic signals at a low field as multiplets (H^p^ δ(^1^H) 8.01 ppm; H^m^ δ(^1^H) 7.73 ppm), which could have been misinterpreted as the formation of a symmetric complex. However, the lack of symmetry of **2** became evident upon inspection of its ^13^C{^1^H}-APT NMR spectrum, which showed two separated doublets (centered at δ(^13^C) 182.9 and 184.0 ppm) for the two carbenic N*C*N atoms. We also clearly observed a CH_2_N fragment centered at δ(^1^H) 5.45 ppm as a diastereotopic pattern,^17^ but interestingly we detected the presence of a singlet at δ(^1^H) 5.97 ppm, unusually at a low field, which correlated with a signal at δ(^13^C) 71.3 ppm in the ^13^C{^1^H}-APT NMR spectrum, which we identified as the fragment “*CH*CO_2_” (Scheme 1). The combination of different NMR techniques, such as ^1^H–^1^H COSY, ^13^C–^1^H HSQC, and ^13^C-APT NMR spectra of **2** (Figures S2–S4), allowed us to identify the structure of **2**, which can be described as the formal addition of CO_2_ to the **=**CH arm in **1**. This assertion was further confirmed by the X-ray study on a single crystal grown from a solution of complex **2** in DMSO-d_6_. The molecular structure of complex **2** is shown in Figure 1, together with the main bond distances and angles.

In compound **2**, the rhodium ion exhibits a square-planar geometry. The CNC* pincer ligand is coordinated in *mer* fashion via pyridine and both imidazolium entities. The fourth coordination site of Rh1 is occupied by a CO ligand, which displays the shortest coordination distance (Rh1–C1, 1.809(2) Å). The CNC* pincer ligand adopts a twist conformation, with pyridine-imidazolium dihedral angles of 50.45(9)° and 53.57(7)°, whereas both imidazolium rings are twisted 67.63(9)° to each other. Thus, insertion of the COO^–^ unit does not seem to distort the coordination environment of the chiral atom C16, as C36 exhibits similar coordination geometries. Both enantiomers of C16 (R and S) are included in the crystal packing.

There is increasing interest in utilizing CO_2_ from different catalytic perspectives.^14^ Besides, there are a number of examples of reactions of lutidine-based transition metal complexes with CO_2_, which happens to react in different ways depending on the pincer structure and the metal involved. For instance, some deprotonated Ru pincer systems are able to activate CO_2_ by forming a C–C bond between the corresponding pincer ligand and the electrophilic carbon atom of CO_2_, with one of the O atoms of the CO_2_ coordinated to Ru,^18^ a reactivity comparable to the [1,3]-addition of CO_2_ to dearomatized PCP Ir^19^ or PNP Re^20^ complexes. There are cases in which CO_2_ reacts directly with the metal, for example, in an aromatized PNP-based Ir complex^21^ and an acridine-based PNP Ni complex,^22^ which also incorporates CO_2_ by bridging Ni pincer moieties.^23^ There is a recent study where CO_2_ inserts into an Ni–R bond in a phenanthroline-based pincer ligand.^24^ As far as we are aware, there is only one structural motif similar to the covalent connection of CO_2_ in complex **2**, as shown in Figure 1, which is based on the addition of CO_2_ to a double deprotonated PNP-based pincer Ni complex, although the reported structure was based on NMR data in solution.^25^

**Scheme 2:**
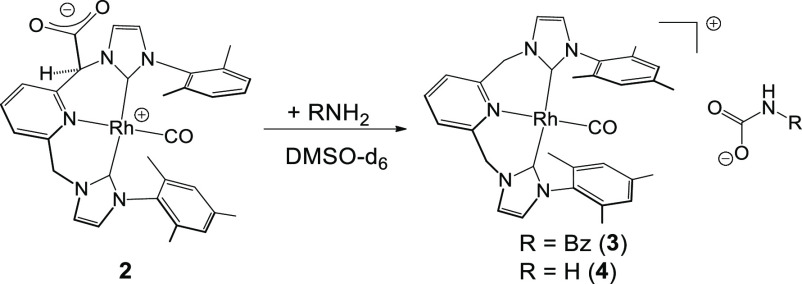
Synthesis of Complexes **3** and **4**

Given the zwitterionic nature of neutral complex [(CNC-CO_2_)^Mes*^Rh(CO)] (**2**), we explored the reactivity of the CO_2_ fragment in **2** with benzylamine (BzNH_2_) and ammonia. NMR monitoring of the reaction of **2** with benzylamine in a 1:1 ratio in DMSO-d_6_ for 5 min quantitatively gave a cationic complex characterized in solution as [(CNC)^Mes^Rh(CO)][HNBzC(*O*)O] (**3**). In a similar way, we monitored the reaction of a solution of **2** in DMSO-d_6_ in a J Young NMR tube under an ammonia atmosphere (2 bar) for 10 min, which cleanly afforded the analogous cationic species, characterized in solution as [(CNC)^Mes^Rh(CO)][H_2_NC(*O*)O] (**4**) (Scheme 2).

Multinuclear NMR characterization in the solution of complexes **3** and **4** was straightforward. A simple comparison of the ^1^H NMR spectra of **3** and **4** allowed us to recognize almost the same pattern of resonances for both species. In fact, the ^1^H NMR spectra of **3** and **4** were almost identical and showed a high degree of symmetry; the pyridinic protons were observed as a triplet (H^p^) and a doublet (H^m^) in a 1:2 ratio. We also observed two well-separated broad signals around δ(^1^H) 5.7 and 5.3 ppm (which correlated with a singlet around δ(^13^C) 54 ppm in their respective ^13^C{^1^H}-APT NMR spectra), corresponding to the CH_2_N arms of the pincer ligand, which indicated that the original **=**CH arm in **2** became protonated in both complexes **3** and **4**. The symmetry of **3** and **4** was also confirmed upon inspection of their respective ^13^C{^1^H}-APT NMR spectra, which showed an unique doublet around 182 ppm (^1^*J*_C–Rh_ = 42 Hz) for the Rh–C(Im) carbons. A deeper comparison of the multinuclear NMR results of **3** and **4** with those reported for the known complex [(CNC)^Mes^Rh(CO)][PF_6_]^15^ allowed us to confirm that species **3^+^** and **4^+^** corresponded to the cation [(CNC)^Mes^Rh(CO)]^+^.

Keeping this situation in mind, we concluded that the anions in species **3** and **4** corresponded to the products of C–N coupling of the CO_2_ fragment in **2** with the incoming amine substrates, that is, carbamates HRNCO_2_^–^ (R = Bz (**3**), H (**4**); Scheme 2). In fact, the CH_2_ fragment of the HBzNCO_2_^–^ anion in **3** was observed at δ(^1^H) 4.70 ppm as a doublet, which correlated with a signal at δ(^13^C) 44.3 ppm in the ^13^C{^1^H}-APT NMR spectrum; moreover, we detected a signal at δ(^13^C) 158.4 ppm, which corresponded to the carboxylic carbon. In the case of complex [(CNC)^Mes^Rh(CO)][H_2_NC(*O*)O] (**4**), we were not able to detect the carbamate anion both in its ^1^H and ^13^C{^1^H}-APT NMR spectra. However, IR data from complexes **3** and **4** showed clearly the carbamoyl carboxylic fragments as strong bands at 1640 and 1635 cm^–1^ for **3** and **4**, respectively, along with sharp peaks terminal for the Rh–CO moieties (1981 cm^–1^ (**3**); 1982 cm^–1^ (**4**)). In addition, the cationic nature of **3** and **4** was confirmed through conductivity measurements in solution (DMSO), which gave values that fit with those shown by 1:1 electrolytes (see the Experimental Section).

The outcome of the aforementioned reactions of **2** with amines contrasts with that of reactions of some carbonyl iridium complexes with primary amines; in particular, complexes [TpIr(CO)] (Tp = tris(pyrazolyl)borate)^26^ or [Ir(CO)(TTP)Cl] (TTP = *meso*-tetra-p-tolylporphyrinato)^27^ react with primary amines and afford the corresponding hydrido carbamoyl complexes. We conclude that formation of cationic species **3** and **4** corresponded to the protonation of the −CH(CO_2_)– arm in **2** from the incoming amine substrates, concomitant with the release of the corresponding carbamate anions through a C–N coupling between the amines and the CO_2_ fragment in **2**.

### Reactivity of [(CNC)^Mes^*Rh(CO)] (**1**) with Terminal Alkynes

**Scheme 3:**
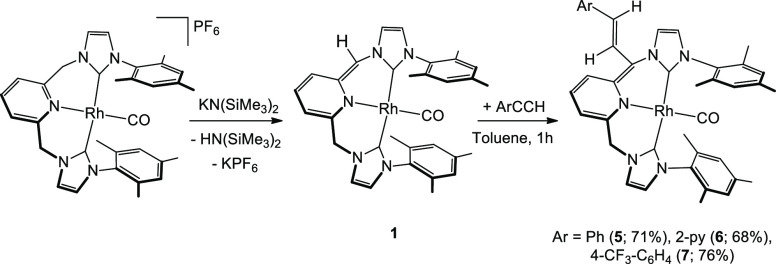
Synthesis of Complexes **5**–**7**

Next, we shifted to the study of the reactivity of the deprotonated compound [(CNC)*Rh(CO)] (**1**) with some unsaturated substrates, more specifically terminal alkynes. In the first place, we reacted complex **1** (formed in situ by reaction of [(CNC)^Mes^Rh(CO)][PF_6_] and KN(SiMe_3_)_2_ in THF, see the Experimental Section) with phenylacetylene, leading to the formation of new species, which was further characterized as the addition product of the alkyne on **1**. Surprisingly, when starting with isolated complex **1**, the reaction with PhCCH did not work properly, a situation that suggested that the presence of a base (HN(SiMe_3_)_2_) was essential for the observed conversion, as confirmed by DFT methods (see below). In this way, in situ formed complex **1** readily added terminal arylacetylenes ArCCH at room temperature (RT) in an unusual way, affording novel complexes characterized as [(CNC-CH**=**CHAr)^Mes^*Rh(CO)] (Ar = Ph (**5**), 2-py (**6**), 4-CF_3_-C_6_H_4_ (**7**); Scheme 3) in good yields.

**Figure 2:**
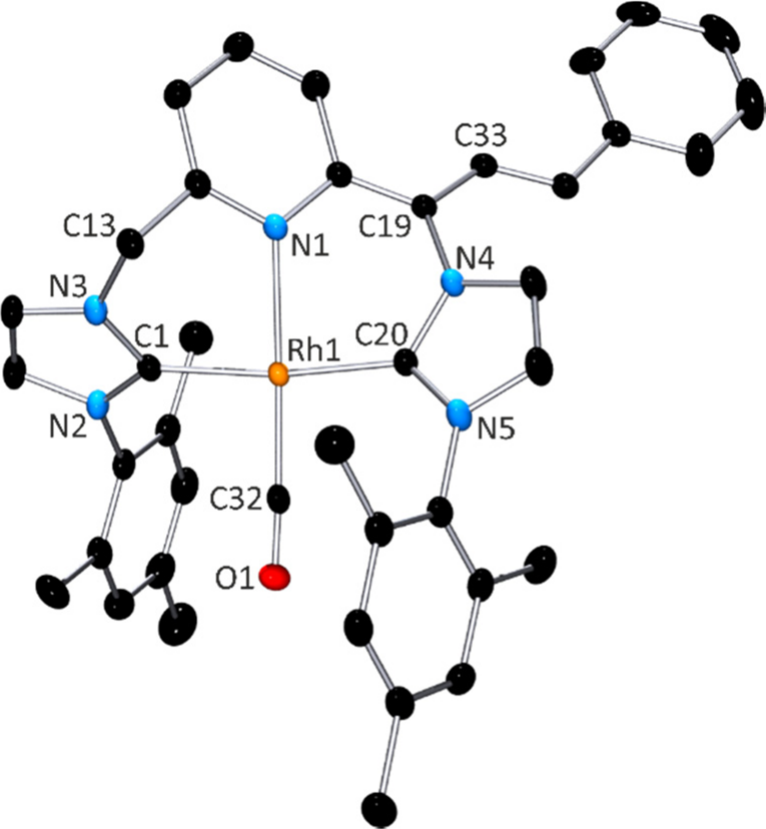
Molecular structure of complex **5**. Hydrogen atoms have been omitted for clarity. Selected distances: Rh1–N1, 2.1285(16) Å; Rh1–C1, 2.0367(19) Å; Rh1–C20, 2.0170(19) Å; and Rh1–C32, 1.821(2) Å. Selected angles: N1–Rh1–C1, 87.16(7)°; N1–Rh1–C20, 84.77(7)°; N1–Rh1–C32, 178.50(8)°; C1–Rh1–C20, 170.95(8)°; C1–Rh1–C32, 91.44(8)°; and C20–Rh1–C32, 96.58(8)°.

As a model, the monitoring of the reaction of complex **1** (prepared in situ) with phenylacetylene in C_6_D_6_ allowed the observation of the clean formation of a new compound, which was fully characterized as complex [(CNC-CH**=**CHPh)^Mes^*Rh(CO)] (**5**; see Scheme 3). The ^1^H NMR spectrum of **5** in C_6_D_6_ showed a complex pattern of resonances that reflected a nonsymmetrical molecule, where the pyridinic group remained dearomatized, defined by three multiplets detected at 7.20, 6.55, and 5.62 ppm, which correlated with signals at 118.1, 129.9, and 105.6 ppm in the ^13^C{^1^H}-APT NMR spectrum, respectively. The most striking feature of the ^1^H NMR spectrum of **5** was the disappearance of the original **=**CH proton from the deprotonated arm in **1**, while the other arm remained as the original methylene backbone. Judging by ^1^H NMR data, it should be mentioned that there is hindered rotation around the mesityl–nitrogen bond, so that the two sides of both rings are distinct. Additionally, we observed two well-separated doublets at δ 7.82 and 6.42 ppm, which correlated respectively with singlets at 124.4 and 109.6 ppm in the ^13^C{^1^H}-APT NMR spectrum of **5**, where the magnitude of the coupling constant (^3^*J*_H–H_ = 15.2 Hz) was consistent with the presence of a *trans* CH**=**CH moiety in complex **5**. A deeper analysis of bidimensional ^1^H–^13^C HSQC, ^1^H–^13^C HMBC, ^1^H–^1^H COSY, and ^1^H-^1^H NOESY of **5** allowed us to confirm that a PhCH**=**CH fragment was covalently connected to the deprotonated **=**CH arm of the CNC* ligand in **1**. This novel structural feature was confirmed by X-ray techniques; the molecular structure of **5** is shown in Figure 2 together with the main geometrical parameters.

As in precursor complex **1**, the *mer* coordination of the CNC* pincer ligand in **5** induced a square planar geometry of the rhodium center. Overall, geometrical features concerning dearomatization of the pyridine ring and Rh–N bond length are close to those of complex **1**.^15^ As shown in Figure 2, the right arm of the molecule in **5** features a covalently bound *trans* PhCH**=**CH– moiety. NMR structural analysis of **5** revealed that the proton attached to the C21 imidazolyl atom in Figure 2 lies right over the electron density from the CH**=**CHPh double bond of the alkene pendant moiety. This situation explains the intense deshielding of proton H21 (δ(^1^H) 7.65 ppm) compared to those of the remaining three **=**CH imidazolyl protons (δ(^1^H) 6.24, 6.17, and 5.89 ppm) observed in the ^1^H NMR spectrum of **5**.

Multinuclear NMR data for complexes **6** and **7** follow the same pattern as those observed for **5**, which indicate that all of them are isostructural. These new complexes **5**–**7** can be described as the products from the formal insertion of the corresponding alkyne molecule into the C(sp^2^)–H bond of the deprotonated arm in **1**. Such transformations involve C–C bond formation and C–H bond activation processes. In an attempt to get insight into the possible mechanism of formation of complex **5**, we carried out in situ deuterium labeling studies with monodeuterated phenylacetylene (PhC≡CD) in combination with complex **1**. The ^2^H NMR spectrum (Figures S29 and S30) of the reaction mixture of **1** and PhC≡CD in a 1:1.5 molar ratio showed after 30 min a broad resonance at δ 7.82 ppm in toluene, which corresponds to the proton attached to the C33 carbon atom in Figure 2. In principle, this observation indicates a formal insertion of the alkyne into the C(sp^2^)–H bond in deprotonated complex **1**; however, upon 3 h of standing, deuterium was observed to be distributed also at the CH_2_N fragment of the molecule at C13, probably due to an H/D exchange with free PhCCD.

The results reported here contrast with those shown by related rhodium/pincer systems. For instance, a lutidine-based PNP highly unsaturated rhodium complex activates terminal acetylenes by forming the corresponding vinylidene derivatives,^28^ whereas a lutidine-based PNP iridium complex rather activates terminal alkynes through oxidative addition of the C(sp)–H bond to iridium, in a process that involves metal–ligand cooperation.^29^

### DFT Study on the Reactivity of [(CNC)^Mes*^Rh(CO)] (**1**) with Terminal Alkynes

**Scheme 4:**
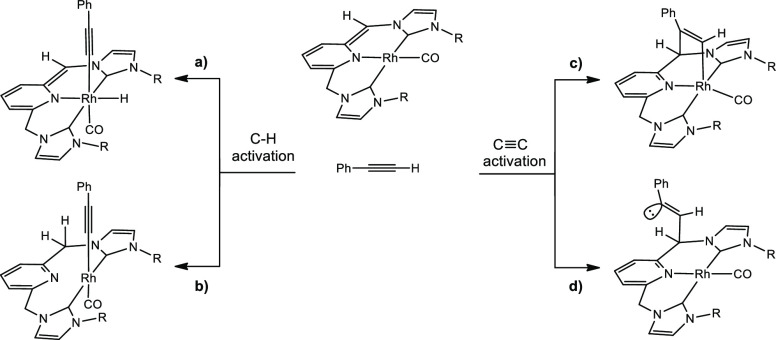
Possible Reaction Pathways of the Reaction between 1 and Phenylacetylene

In order to shed light into the obtained results, a DFT study on the reactivity of **1** with phenylacetylene has been carried out. The full ligand CNC^Mes^* has been considered in the calculations (R = mesityl). Based on preliminary studies, we propose four possible pathways involving respective C–H and C≡C triple bond activations of phenylacetylene mediated by complex **1** (Scheme 4).

**Figure 3:**
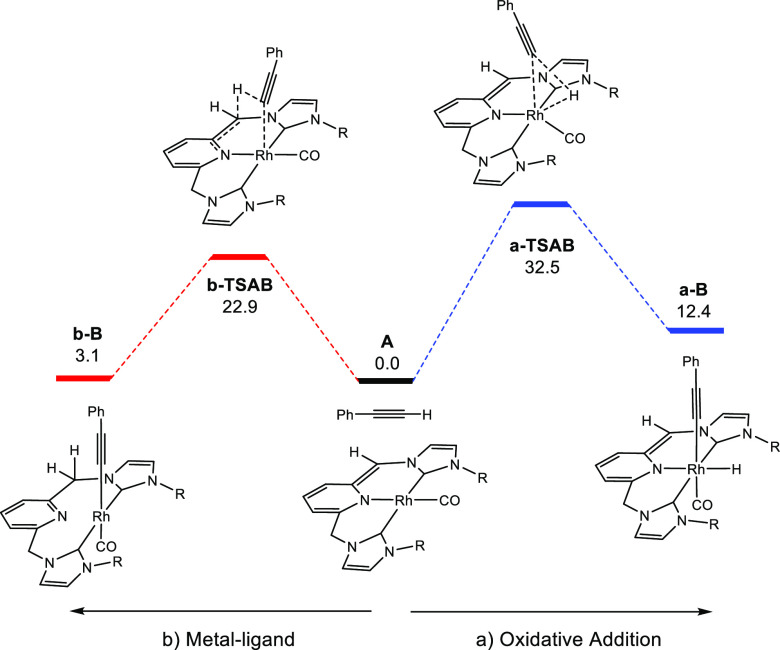
DFT energetic profiles for C–H and C–C bond activations promoted by complex **1** (energies in kcal mol^–1^).

The results of alkyne activation through paths (a) and (b) are shown in Figure 3. Pathway (a) is based on the oxidative addition of the C–H bond to the metal, which presents an energetic barrier of 32.5 kcal mol^–1^, with the resulting Rh(III) product being exergonic (12.4 kcal mol^–1^). Path (b) depicts the activation of the C–H bond of the alkyne via metal–ligand cooperation, which occurs through an affordable energetic barrier of 22.9 kcal mol^–1^ characterized by **b-TSAB**; this transition state further leads to a square planar Rh(I) alkynyl intermediate **b-B**, where the pyridine moiety is no longer coordinated to the metal.

**Figure 4:**
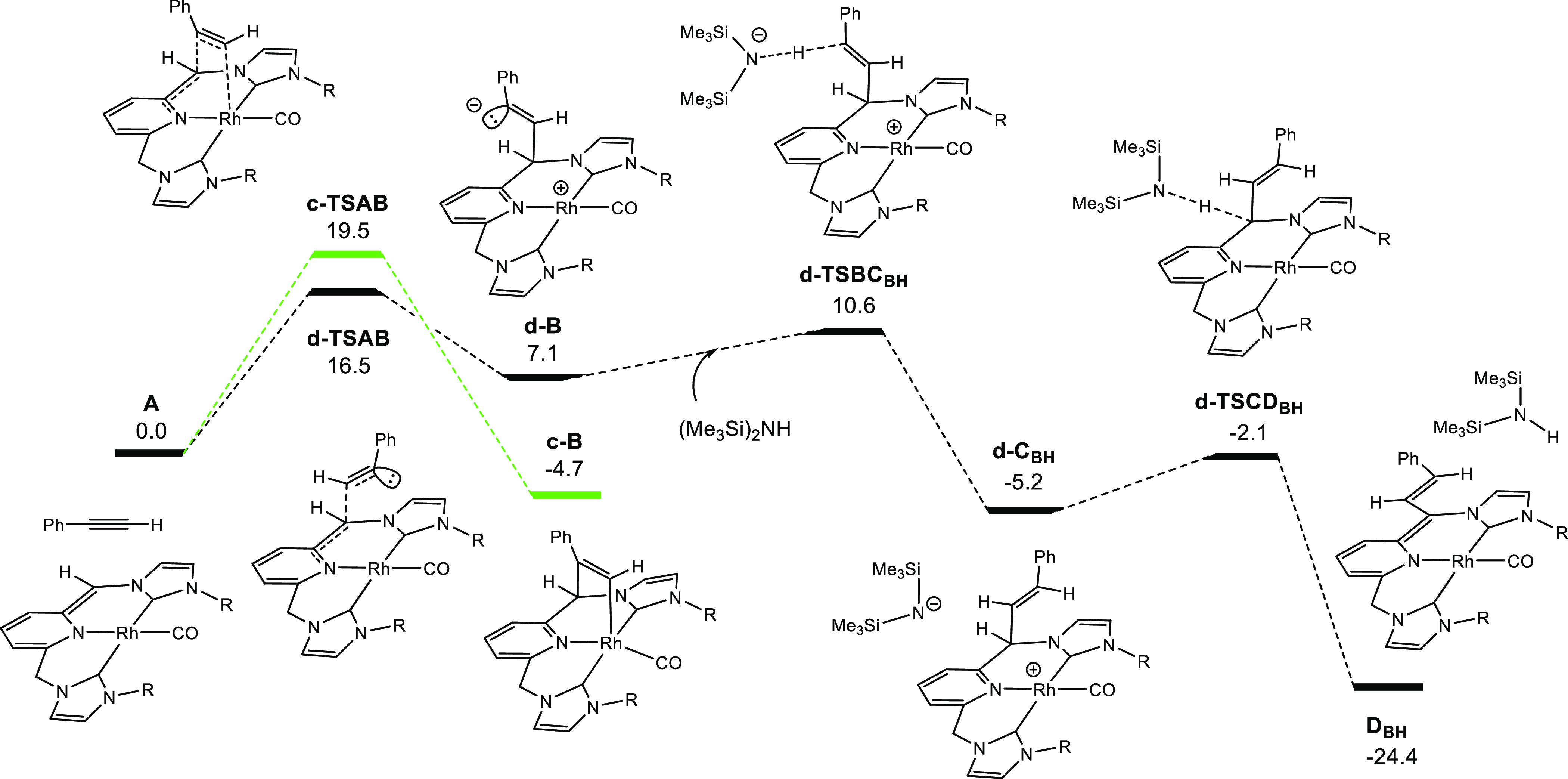
DFT energetic profiles, including solvent corrections for toluene, for C≡C bond activation by complex **1** following paths (c) and (d), energies in kcal mol^–1^.

The DFT analyses for the C≡C bond activation by complex **1** are illustrated in Figure 4. Pathway (c) (in green) corresponds to metal–ligand cooperative (MLC) activation of the triple bond, characterized by transition state **c-TSAB** showing an energetic barrier of 19.5 kcal mol^–1^. Both C–C and Rh–C bonds form in a concerted manner via MLC leading to metallacycle **c-B**, which is exergonic (−4.7 kcal mol^–1^). It should be noted that the terminal carbon of the alkyne covalently connects to the metal in spite of the large steric congestion due to the proximity of the voluminous mesityl groups of the bis-carbene ligands. Within this line, a similar mechanism has been calculated for the other possible orientation of phenylacetylene, but it could not be found due to the steric repulsion between the phenyl group of the alkyne and the mesityl ligands.

**Figure 5:**
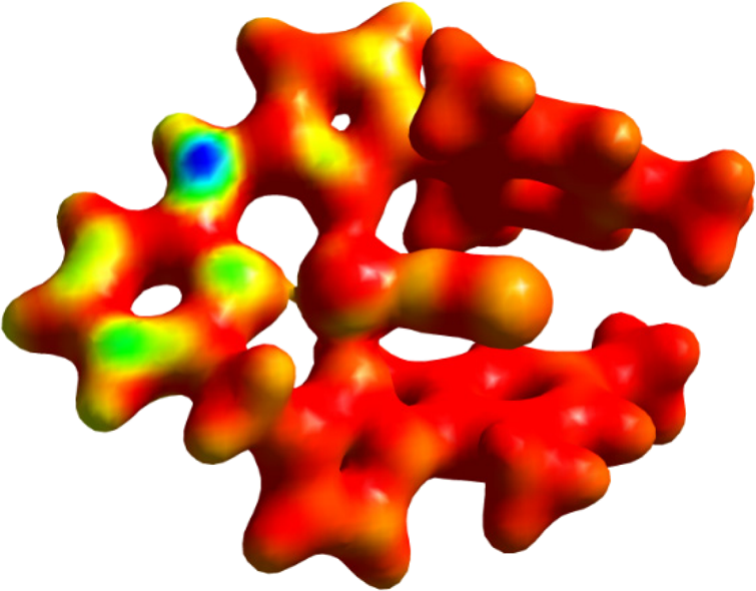
Fukui minus function (*f*-). The sites susceptible of electrophilic attack in complex **1** are colored in blue. Red color indicates zero values. Density isocontour value of 0.07 a.u.

Alternatively, path (d) involves an electrophilic attack of the terminal carbon of the alkyne to the deprotonated **=**CH arm of the CNC^Mes^* ligand by forming a new C–C bond. This step leads to transient secondary carbanion species, namely, intermediate **d-B**, which can be stabilized by resonance from the adjacent phenyl group. Geometrical characterization and a representation of the HOMO of **d-B** are shown in Figure S30. The activation energy for this step is 16.5 kcal mol^–1^ and therefore is the lowest energetic barrier of the four proposed pathways studied (pathways a–d). This unexpected mechanism can be rationalized by inspection of the Fukui function for the removal of an electron from complex **1** shown in Figure 5, which reveals the sites on a molecule that are susceptible for electrophilic attack.^30^ The blue spot at the deprotonated **=**CH arm indicates that electron density is susceptible for an electrophilic attack at the carbon atom. The intermediate formed, **d-B**, is exergonic (7.1 kcal mol^–1^), and it can be stabilized by the protic base present in the reaction media (HN(SiMe_3_)_2_). In this situation, the approximation and further proton transfer from an HN(SiMe_3_)_2_ molecule takes place via **d-TSBC_BH_**, presenting an energetic barrier of 10.6 kcal mol^–1^ and leading to **d-C_BH_**. Finally, the base can abstract the proton through **d-TSCD_BH_** leading to product **D_BH_**, which can conjugate the double bonds over an extended π system, and it is strongly exergonic (−24.4 kcal mol^–1^).

## Conclusions

In this contribution, we have shown the reactivity of a carbonyl rhodium(I) complex stabilized with a deprotonated CNC^Mes^* pincer ligand. The presence of an “**=**CH” arm in the complex induces a high nucleophilic character, so reactivity with electrophiles was expected. In fact, the carbonyl complex reacted with CO_2_ (g) to yield a new zwitterionic complex, which displays a CO_2_ moiety covalently linked to the pincer skeleton in the former “**=**CH” arm. This CO_2_ zwitterionic adduct readily reacted with nucleophiles such primary amines (benzylamine and ammonia), which gave the protonated, cationic Rh carbonyl compound, where the corresponding anions were the corresponding carbamates [HNRCO_2_]^−^ (R = Bz, H), which are the products of the C–N coupling between the CO_2_ fragment and the respective incoming amine. We further explored the reactivity of the deprotonated carbonyl Rh(I) complex with selected terminal alkynes ArC≡CH (Ar = Ph, 2-py, 4-CF_3_C_6_H_4_), which cleanly afforded new complexes, which showed the incoming alkyne covalently connected to the CNC ligand. Both deuterium-labeling experiments and DFT studies show these compounds to be the formal products of the insertion of the incoming alkyne into the **=**C–H bond of the arm. In particular, DFT studies showed an MLC mechanism, where the **=**CH arm undergoes a nucleophilic attack on the alkyne affording a carbanion intermediate. The presence of a protic base is necessary as it acts as proton shuttle to achieve the formation of the final product. It should be stressed that the nucleophilicity of the **=**CH arm of the ligand encompasses the change of the character of the N–Rh bond (amido/amino), setting in this way the basis for an MLC mechanism in which the metal does not change its oxidation state during the whole transformation.

## Experimental Section

### Scientific Equipment

C, H, and N analyses were carried out in a Perkin-Elmer 2400 Series II CHNS/O analyzer. IR spectra of solid samples were recorded with a Perkin-Elmer 100 FT-IR spectrometer (4000–400 cm^–1^) equipped with attenuated total reflectance. ^1^H, ^31^P{^1^H}, and ^13^C{^1^H} NMR spectra were recorded on Bruker Avance 300 (300.13, 121.42, and 75.48 MHz) and Bruker Avance 400 (400.16, 161.99, and 100.61 MHz) spectrometers. NMR chemical shifts are reported in ppm relative to tetramethylsilane and are referenced to partially deuterated solvent resonances. Coupling constants (*J*) are given in hertz. Spectral assignments were achieved by combination of ^1^H–^1^H COSY, ^1^H–^1^H NOESY, ^13^C{^1^H}-APT, ^1^H–^13^C HSQC, and ^1^H–^13^C HMBC NMR experiments. ESI^+^ mass spectra were obtained with a Bruker MICROFLEX spectrometer using 1,8-dihydroxy-9,10-dihydroanthracen-9-one (DIT, dithranol) as the matrix. GC–MS analyses were recorded on an Agilent 5973 mass selective detector interfaced to an Agilent 6890 series gas chromatograph system, using an HP-5MS 5% phenyl methyl siloxane column (30 m × 0.250 mm with a 0.25 μm film thickness).

### Synthesis

All experiments were carried out under an atmosphere of argon using Schlenk and dry box techniques. Solvents were distilled immediately prior to use from the appropriate drying agents or obtained from a solvent purification system (Innovative Technologies). Oxygen-free solvents were employed throughout. CD_2_Cl_2_ and (CD_3_)_2_CO were dried using activated molecular sieves, while C_6_D_6_ and toluene-d_8_ were dried over a solid Na/K amalgam. Carbon monoxide was purchased from Air Liquid. Compounds [(CNC)^Mes^Rh(CO)][PF_6_] and **1** were prepared according to published procedures.^15^ Deuterated phenylacetylene (PhCCD) was prepared by following a recent synthetic procedure.^31^

#### In Situ Formation of [(CNC-CO_2_)^Mes^*Rh(CO)] (**2**)

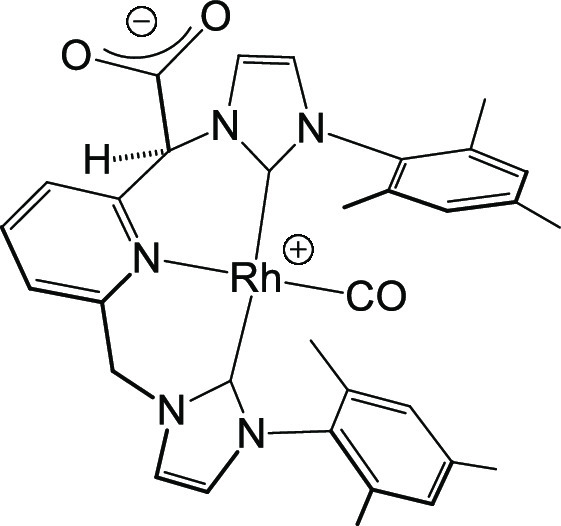



In an oxygen-free J Young pressure NMR tube (Wilmad) were suspended crystals of **1** (25 mg, 0.04 mmol) in DMSO-d_6_ (0.4 mL). The resulting red mixture was pressurized with carbon dioxide (5 bar), affording a yellow solution, which was immediately subjected to a full NMR characterization, allowing observation of the complete and clean formation of complex **2**. ^1^H NMR (300 MHz, DMSO-d_6_, 298 K): δ 8.01 (m, 1H; H^p^ py), 7.73 (m, 2H; H^m^ py), 7.21 (d, ^3^*J*_H–H_ = 1.3 Hz, 2H), 7.14 (d, ^3^*J*_H–H_ = 1.4 Hz, 2H) (**=**CH Im), 6.92 (1H), 6.89 (1H), 6.85 (1H), 6.84 (1H) (br; CH Mes), 5.97 (s, 1H; C*H*CO_2_), 5.66 (d, ^3^*J*_H–H_ = 14.2 Hz; CH_2_N), 5.24 (d, ^3^*J*_H–H_ = 14.2 Hz; CH_2_N), 2.23, 2.20, 1.94, 1.92, 1.87, 1.82 (Me Mes). ^13^C{^1^H} NMR (75 MHz, DMSO-d_6_, 298 K): δ 191.5 (d, ^1^*J*_C–Rh_ = 80.1 Hz; CO), 184.0 (d, ^1^*J*_C–Rh_ = 42 Hz; Rh–C Im), 182.9 (d, ^1^*J*_C–Rh_ = 42 Hz; Rh–C Im), 166.0 (CO_2_), 156.1, 154.1 (CN py), 139.7 (s; C^p^ py), 138.5, 138.0, 136.9, 136.6, 135.9, 135.7, 135.3, 134.9 (C_q_ arom.), 129.2, 129.0, 128.9, 128.8 (CH Mes), 126.7 (s; C^m^ py), 122.2, 121.1 (**=**CH Im), 71.3 (*C*HCO_2_), 54.6 (d, ^3^*J*_C–Rh_ = 33 Hz; CH_2_N), 21.1, 21.0, 19.0, 18.9, 18.1, 17.9 (Me Mes). Mass calcd for C_33_H_32_N_5_O_3_Rh: 649.1560; mass calcd for C_32_H_32_N_5_ORh: 605.5349; HRMS (ESI+): *m*/*z* 606.1740 (100%; M^+^ – CO_2_ + 1H). IR (ATR): υ(CO_2_) 1625 cm^–1^, υ(Rh–CO) 1982 cm^–1^.

#### In Situ Formation of [(CNC)^Mes^Rh(CO)][HBzNC(*O*)O] (**3**)

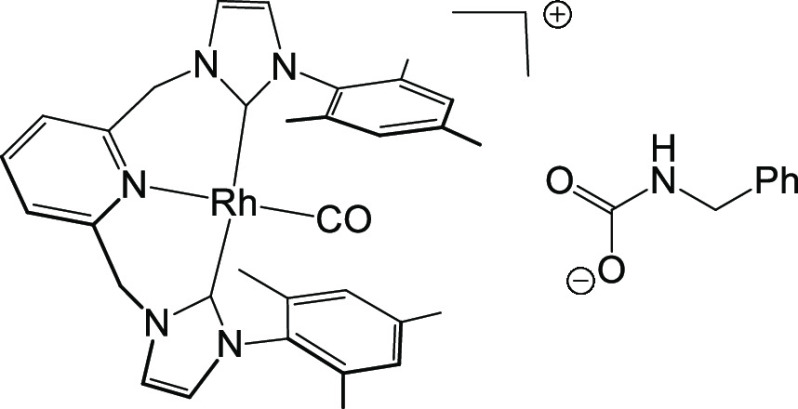



An oxygen-free J Young pressure NMR tube (Wilmad) was charged with complex **2** (25 mg, 0.04 mmol), dissolved in DMSO-d_6_ (0.4 mL), and pressurized with CO_2_ (5 bar). Then, neat benzylamine was added (4.4 μL, 0.04 mmol) with a microsyringe, and upon 5 min, the solution turned dark-yellow, showing the complete transformation into complex **6** by NMR techniques. ^1^H NMR (300 MHz, DMSO-d_6_, 298 K): δ 8.18 (t, ^3^*J*_H–H_ = 7.7 Hz, 1H; H^p^ py), 7.85 (d, ^3^*J*_H–H_ = 7.8 Hz, 2H; H^m^ py), 7.76 (d, ^3^*J*_H–H_ = 1.8 Hz, 2H), 7.34 (d, ^3^*J*_H–H_ = 1.8 Hz, 2H) (=CH Im), 7.22–7.30 (set of m, 6H; *Ph*CH_2_N + N-*H*), 6.95 (br, 4H; CH Mes), 5.64 (br, 2H), 5.45 (br, 2H) (CH_2_N), 4.70 (d, ^3^*J*_H–H_ = 6.0 Hz, 2H; PhC*H*_2_N), 2.25 (s, 6H), 1.90 (s, 12H) (Me Mes). ^13^C {^1^H}-APT NMR (400 MHz, DMSO-d_6_, 298 K): δ 190.4 (d, ^1^*J*_C–Rh_ = 80 Hz; CO), 182.5 (d, ^1^*J*_C–Rh_ = 42 Hz; Rh–C Im), 158.4 (*C*O_2_NHBz), 156.1 (C^o^ py), 141.9 (C^p^ py), 141.2 (C^ipso^*Ph*CH_2_N), 138.6, 135.9 (*C*-Me Mes), 135.4 (br; N-*C* Mes), 129.0 (br; CH Mes), 128.5 (C^o^), 127.3 (C^m^), 126.9 (C^p^) (*Ph*CH_2_N), 125.2 (C^m^ py), 122.8, 122.7 (**=**CH Im), 54.6 (CH_2_N), 44.3 (s; Ph*C*H_2_N). 21.0 (s), 18.3 (br) (Me Mes). Mass calcd for C_32_H_33_N_5_ORh: 606.1740; HRMS (ESI+): *m*/*z* 606.1740 (100%; M^+^). IR (ATR): υ(CO_2_) 1640 cm^–1^, υ(CO) 1981 cm^–1^. Λ_M_ (Ω^–1^ cm^2^ mol^–1^): 168.7 (DMSO, 2.0 × 10^–4^ M).

#### In Situ Formation of [(CNC)^Mes^Rh(CO)][H_2_NC(*O*)O] (**4**)

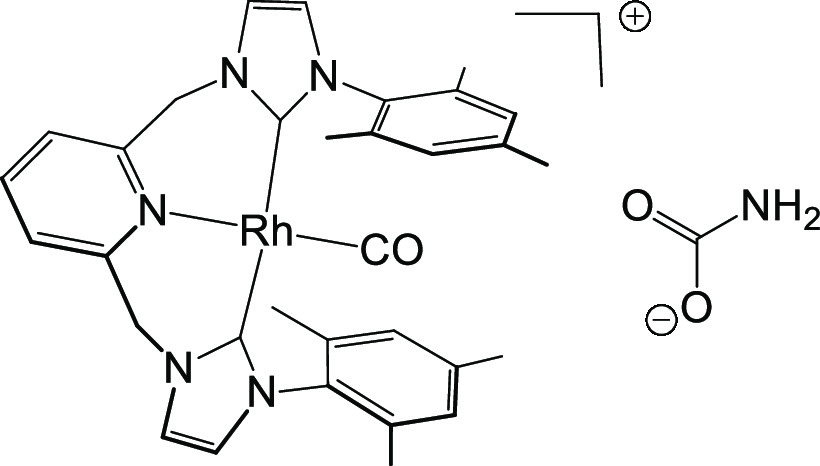



An oxygen-free J Young pressure NMR tube (Wilmad) was charged with complex **2** (25 mg, 0.04 mmol), dissolved in DMSO-d_6_ (0.4 mL), and pressurized with CO_2_ (5 bar). The resulting solution was frozen in liquid nitrogen for a few seconds, and then, the argon atmosphere was removed and replaced with gaseous ammonia (2 bar). Upon 10 min of warming, NMR measurements showed the complete and clean transformation into complex **4**. ^1^H NMR (300 MHz, DMSO-d_6_, 293 K): δ 8.17 (t, ^3^*J*_H–H_ = 7.7 Hz, 1H; H^p^ py), 7.86 (d, ^3^*J*_H–H_ = 7.8 Hz, 2H; H^m^ py), 7.75 (br, 2H), 7.29 (br, 2H) (**=**CH Im), 6.92 (br, 4H; CH Mes), 5.69 (br, 2H), 5.48 (br, 2H) (CH_2_N), 2.23 (s, 6H), 1.88 (s, 12H) (CH_3_ Mes). ^13^C {^1^H}-APT NMR (300 MHz, DMSO-d_6_, 293 K): δ 190.7 (d, ^1^*J*_C–Rh_ = 80 Hz; CO), 182.8 (d, ^1^*J*_C–Rh_ = 42 Hz; Rh–C Im), 156.1 (C^o^ py), 141.9 (C^p^ py), 138.6, 135.9 (*C*-Me Mes), 135.4 (br; N-*C* Mes), 128.9 (br; CH Mes), 125.3 (C^m^ py), 122.6, 122.5 (**=**CH Im), 54.5 (br; CH_2_N), 20.9 (s), 18.0 (br) (Me Mes). Mass calcd for C_32_H_33_N_5_ORh: 606.1740; HRMS (ESI+): m/z 606.1740 (100%; M^+^). IR (ATR): υ(CO_2_): 1635 cm^–1^, υ(CO) 1982 cm^–1^. Λ_M_ (Ω^–1^ cm^2^ mol^–1^): 114.9 (DMSO, 2.0 × 10^–4^ M).

#### Synthesis of [(CNC-CH**=**CHPh)^Mes^*Rh(CO)] (**5**)

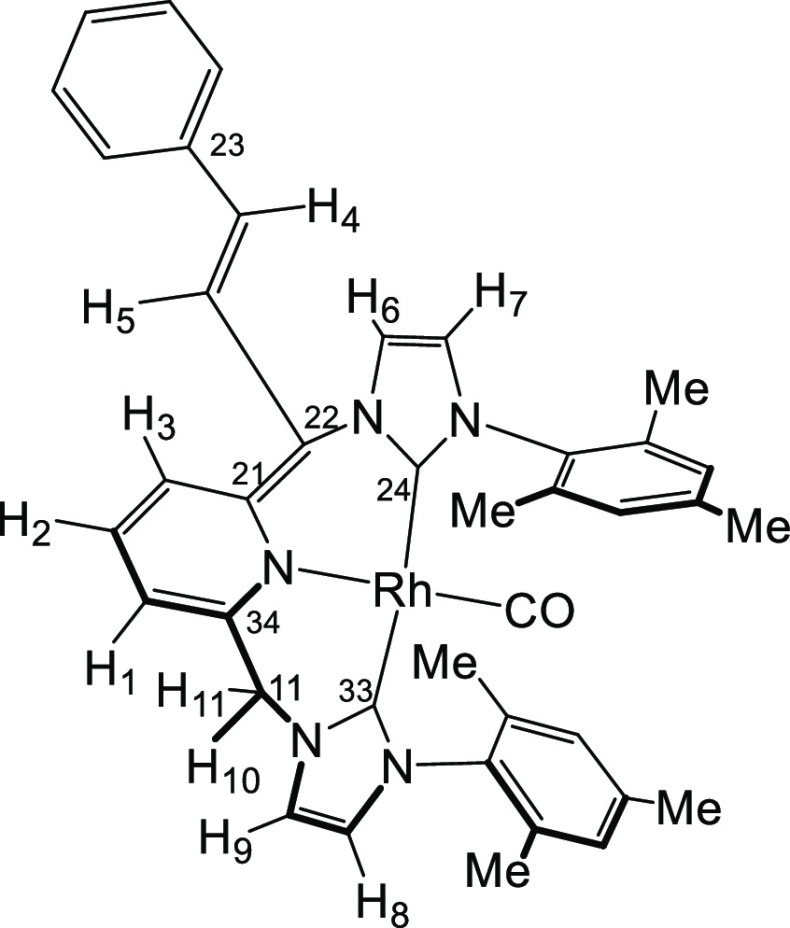



To a yellow solution of complex [(CNC)^Mes^Rh(CO)][PF_6_] (0.10 g, 0.13 mmol) in toluene (5 mL), solid KN(SiMe_3_)_2_ (27 mg, 0.13 mmol) was added, and the resulting deep red suspension was stirred for 1 h. The mixture was filtered via a cannula, and then, pure phenylacetylene was added via syringe (13 μL, 0.12 mmol), affording a dark brown solution, which was stirred for 3 h. The volume was concentrated to ca. 2 mL by vacuum, and the addition of hexanes led to the precipitation of a dark brown solid, which was subsequently washed with hexanes and then dried under vacuum. Yield: 60 mg (71%). ^1^H NMR (300 MHz, C_6_D_6_, 298 K): δ 7.82 (d, ^3^*J*_H–H_ = 15.2 Hz, 1H; CC*H*^5^ = CHPh), 7.65 (d, ^3^*J*_H–H_ = 1.9 Hz, 1H; **=**CH^6^ Im), 7.44 (m, 1H), 7.26 (m, 1H) (Ph), 7.20 (d, ^3^*J*_H–H_ = 9.0 Hz, 1H; H^3^), 7.11 (m, 1H), 7.01 (m, 2H) (Ph), 6.76 (br, 1H), 6.68 (br, 1H), 6.59 (br, 1H) (CH Mes), 6.55 (dd, ^3^*J*_H–H_ = 9.0 Hz, ^3^*J*_H–H_ = 6.2 Hz, 1H; H^2^), 6.42 (d, ^3^*J*_H–H_ = 15.2 Hz, 1H; CCH**=**C*H*^4^Ph), 6.33 (br, 1H; CH Mes), 6.24 (d, ^3^*J*_H–H_ = 1.9 Hz, 1H) (H^9^), 6.17 (d, ^3^*J*_H–H_ = 1.9 Hz, 1H) (H^7^), 5.89 (d, ^3^*J*_H–H_ = 1.9 Hz, 1H) (H^8^) (**=**CH Im), 5.62 (d, ^3^*J*_H–H_ = 6.2 Hz, 1H; H^1^), 4.68 (d, ^3^*J*_H–H_ = 12.6 Hz, 1H) (H^11^), 3.82 (d, ^3^*J*_H–H_ = 12.6 Hz, 1H) (H^10^) (CH_2_N), 2.35 (s, 3H), 2.07 (s, 3H), 1.98 (s, 3H), 1.88 (s, 3H), 1.86 (s, 3H), 1.74 (s, 3H) (Me). ^13^C{^1^H} NMR (75 MHz, C_6_D_6_, 298 K): δ 193.1 (d, ^1^*J*_C–Rh_ = 77 Hz; CO), 189.9 (d, ^1^*J*_C–Rh_ = 43 Hz; C^33^), 182.2 (d, ^1^*J*_C–Rh_ = 42 Hz; C^24^) (Rh–C Im), 152.2 (CH_2_*C*^34^N), 150.6 (N_py_*C*^21^ = CN), 142.8 (C^ipso^ Ph) (C^23^), 138.6, 138.5, 138.2, 137.3, 137.1, 137.0, 136.9, 135.0 (C_q_), 130.7, 130.6 (CH Mes), 129.9 (C^2^), 129.6, 129.5 (CH Mes), 129.4, 129.2, 129.1, 125.0 (Ph), 124.4 (C*C*^5^H**=**CHPh), 123.5 (Ph), 123.3 (C^6^), 121.5 (C^8^), 120.0 (C^9^), 119.9 (C^7^) (**=**CH Im), 118.1 (C^3^), 109.6 (CCH**=***C*^4^HPh), 107.6 (*C*^22^CH**=**CHPh), 105.6 (C^1^), 58.6 (C^11^H_2_N), 21.7, 21.5, 20.1, 19.6, 18.4, 18.3 (Me Mes). Mass calcd for C_40_H_38_N_5_ORh: 707.2131; HRMS (ESI^+^): *m*/*z* 708.2210 (100%; M^+^ + 1H). IR (ATR): υ(CO) 1974 cm^–1^.

#### Synthesis of [(CNC-CH**=**CH-2-C_5_H_4_N)^Mes^*Rh(CO)] (**6**)

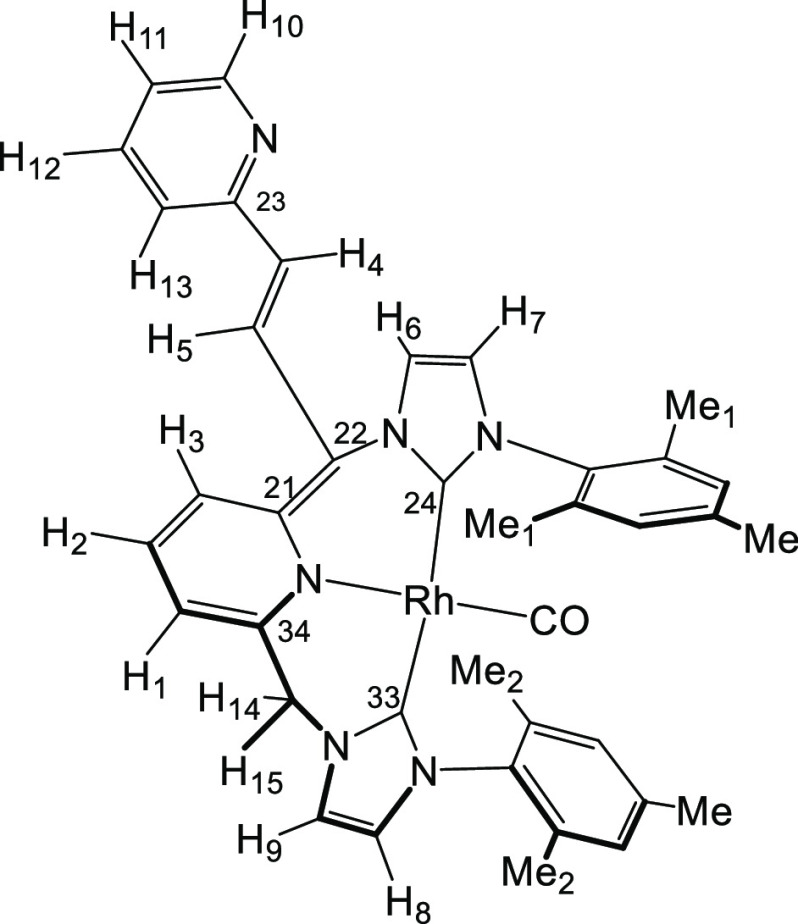



A solution of [(CNC)^Mes^Rh(CO)][PF_6_] (0.10 g, 0.13 mmol) in toluene (5 mL) was treated with solid KN(SiMe_3_)_2_ (27 mg, 0.13 mmol), forming a deep red solution that was stirred at RT for 1 h. This was filtered to eliminate the formed salt, and then, pure 2-ethynylpyridine was added via a syringe (19 μL, 0.12 mmol), affording a dark violet mixture, which was stirred at RT for 1 h. Then, the solvent was removed by vacuum; the remaining powder was washed with hexanes and then dried under vacuum. Yield: 58 mg (68%). ^1^H NMR (300 MHz, C_6_D_6_, 298 K): δ 9.02 (d, ^3^*J*_H–H_ = 14.4 Hz, 1H; CC*H*^5^ = CHPy), 8.65 (dd, ^3^*J*_H–H_ = 4.8 Hz, ^4^*J*_H–H_ = 1.4 Hz 1H; H^10^), 7.69 (d, ^3^*J*_H–H_ = 1.9 Hz, 1H; **=**CH^6^ Im), 7.51 (d, ^3^*J*_H–H_ = 9 Hz, 1H; H^3^), 7.12 (m, 1H; H^12^), 6.83 (d, ^3^*J*_H–H_ = 8.4 Hz, 1H; H^13^), 6.76 (m, 1H), 6.67 (m, 1H), 6.58 (m, 1H) (CH Mes), 6.55 (m, 1H; H^11^), 6.47(m, 1H; H^2^), 6.41 (d, ^3^*J*_H–H_ = 14.4 Hz, 1H; CCH**=**C*H*^4^Py), 6.31 (m, 1H; CH Mes), 6.22 (d, ^3^*J*_H–H_ = 1.9 Hz, 1H; **=**CH^9^ Im), 6.16 (d, ^3^*J*_H–H_ = 1.9 Hz, 1H; **=**CH^7^ Im), 5.86 (d, ^3^*J*_H–H_ = 1.9 Hz, 1H; **=**CH^8^ Im), 5.62 (dd, ^3^*J*_H–H_ = 6.3 Hz, ^4^*J*_H–H_ = 0.9 Hz, 1H; H^1^), 4.68 (d, ^3^*J*_H–H_ = 12.5 Hz, 1H; H^14^), 3.82 (d, ^3^*J*_H–H_ = 12.5 Hz, 1H; H^15^) (CH_2_N), 2.31 (s, 3H), 1.85 (s, 3H) (Me^1^), 2.06 (s, 3H), 1.98 (s, 3H) (Me^p^), 1,79 (s, 3H), 1.73 (s, 3H) (Me^2^) (Me Mes). ^13^C{^1^H} NMR (75 MHz, C_6_D_6_, 298 K): δ 192.0 (d, ^1^*J*_C–Rh_ = 74 Hz; CO), 189.6 (d, ^1^*J*_C–Rh_ = 42 Hz; C^33^), 182.3 (d, ^1^*J*_C–Rh_ = 42 Hz; C^24^) (Rh–C Im), 160.9 (C^ipso^ py) (C^23^), 153.2 (N_py_*C*^21^ = CN), 151. 8 (CH_2_*C*^34^N), 150.2 (C^o^ Py) (C^10^), 138.5, 138.3 (C_q_ Mes), 137.1, 134.8 (C_q_N Mes), 137.9, 136.8, 137.0, 136.6 (C_q_ Mes), 135.7 (C^12^), 131.4 (C^2^), 130.4, 129.4, 129.3, 129.0 (CH Mes), 127.0 (*C*^5^H**=**CHPh), 123.3 (C^6^) 121.4 (C^8^), 119.9 (C^9^), 119.4 (C^7^) (**=**CH Im), 120.1 (C^13^), 119.8 (C^3^), 116.6 (C^11^), 107.8 (C^22^), 107.3 (C^1^), 106.0 (CCH**=***C*^4^HPh), 58.2 (d, ^4^*J*_C–Rh_ = 1.6 Hz) (CH_2_N), 21.3, 21.4 (Me^p^ Mes), 19.8, 19.2, 18.2, 18.1 (Me^o^ Mes). Mass calcd for C_39_H_37_N_6_ORh: 708.2084; HRMS (ESI^+^): *m/z* 708.2133 (100%; M^+^ + 1H). IR (ATR): υ(CO) 1984 cm^–1^.

#### Synthesis of [(CNC-CH**=**CH-4–C_6_H_4_CF_3_)^Mes^*Rh(CO)] (**7**)

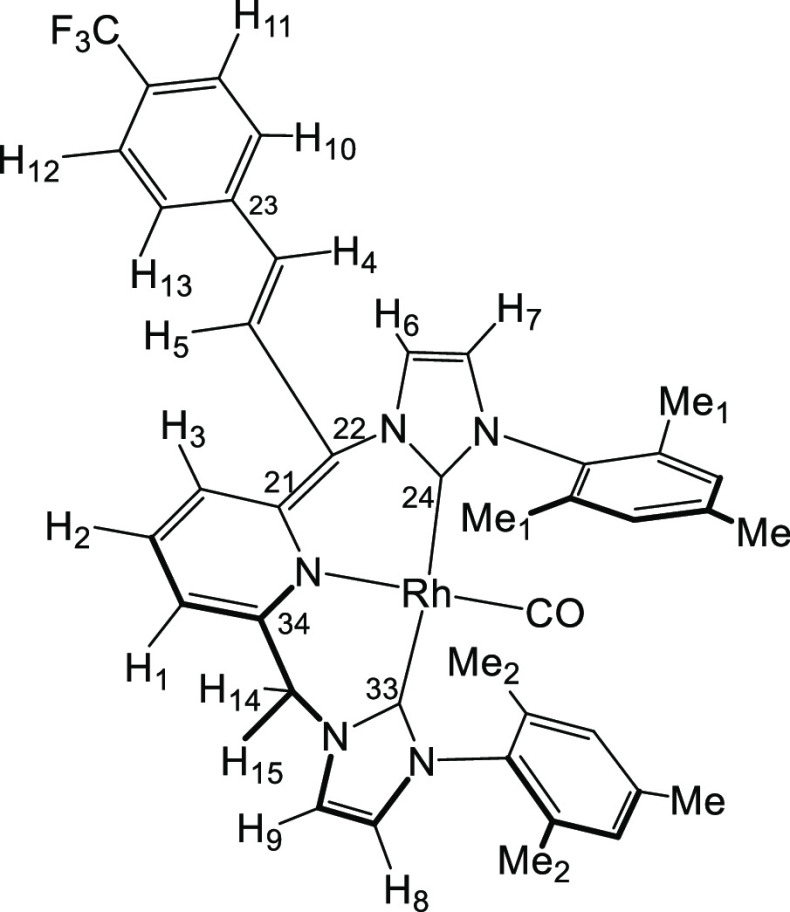



To a yellow solution of [(CNC)^Mes^Rh(CO)][PF_6_] (0.10 g, 0.13 mmol) in toluene (5 mL), solid KN(SiMe_3_)_2_ (27 mg, 0.13 mmol) was added, and the resulting deep red suspension was stirred for 1 h, affording a deep red solution. The mixture was filtered via a cannula, and then, pure 4-trifluoromethylphenylacetylene was added via a syringe (20 μL, 0.12 mmol), affording a dark violet mixture, which was stirred at RT for 1 h. Then, the solvent was removed by vacuum; the remaining powder was washed with hexanes and then dried under vacuum. Yield: 70 mg (76%). ^1^H NMR (300 MHz, C_6_D_6_, 298 K): δ 7.86 (d, ^3^*J*_H–H_ = 14.8 Hz, 1H; CC*H*^5^ = CHAr), 7.67 (d, ^3^*J*_H–H_ = 1.9 Hz, 1H; **=**CH^6^ Im), 7.59 (m, 1H) 7.56 (m, 1H) (H^10^, H^13^; CH^m^ Ar), 7.32 (m, 1H; H^3^), 7.31 (m, 1H) 7.27 (m, 1H) (H^11^, H^12^; CH^o^ Ar), 6.86 (m, 1H) 6.79 (m, 1H) (CH Mes), 6.71 (m, 1H; H^2^), 6.69 (m, 1H; CH Mes), 6.41 (m, 1H; CH Mes), 6.31 (d, ^3^*J*_H–H_ = 14.8 Hz, 1H; CCH**=**C*H*^4^Ar), 6.29 (d, ^3^*J*_H–H_ = 1.9 Hz, 1H; **=**CH^9^ Im) 6.28 (d, ^3^*J*_H–H_ = 1.9 Hz, 1H; **=**CH^7^ Im), 5.96 (d, ^3^*J*_H–H_ = 1.9 Hz, 1H; **=**CH^8^ Im), 5.77 (dd, ^3^*J*_H–H_ = 6.3 Hz, ^4^*J*_H–H_ = 0.7 Hz, 1H; H^1^), 4.78 (d, ^3^*J*_H–H_ = 12.9 Hz, 1H; H^14^), 3.88 (d, ^3^*J*_H–H_ = 12.9 Hz, 1H; H^15^) (CH_2_N), 2.42 (s, 3H) 1.96 (s, 3H) (Me^1^) 2.17 (s, 3H) 2.08 (s, 3H) (Me^p^) 1.93 (s, 3H) 1.82 (s, 3H) (Me^2^) (Me Mes). ^13^C{^1^H} NMR (75 MHz, C_6_D_6_, 298 K): δ 192.6 (d, ^1^*J*_C–Rh_ = 78.2 Hz; CO), 189.3 (d, ^1^*J*_C–Rh_ = 42 Hz; C^33^), 182.6 (d, ^1^*J*_C–Rh_ = 42 Hz; C^24^), 152.6, (N_py_*C*^21^ = CN) 152.1 (CH_2_*C*^34^N) 146.6 (C^ipso^ py, C^23^), 138.6, 138.5 (C^p^_q_ Mes), 137.8, 136.55 (C_q_N Mes), 137.0, 136.8, 136.7, 134.8 (C^o^_q_ Mes), 131.5 (C^8^), 130.4, 129.5, 129.4, 129.1 (CH Mes), 128.8, 126.2, 123.7, 118.7 (C^10^, C^11^, C^12^, C^13^; Ar), 126.6 (q, ^2^*J*_C–F_ = 1.36 Hz; *C*CF_3_), 126.3 (*C*^5^H**=**CHPh), 126.1 (q, ^1^*J*_C–F_ = 4 Hz; CF_3_), 123.1 (C^6^), 121.4 (C^8^), 119.9, (C^9^) 119.7 (C^7^), 107.5 (C^22^), 107.2 (C^1^), 104.9 (CCH**=***C*^4^HPh), 58.2 (CH_2_N), 21.4, 21.3 (Me^p^ Mes), 19.8, 19.3, 18.2, 18.1 (Me^o^ Mes). Mass Calcd for C_41_H_37_F_3_N_5_ORh: 775.2205; HRMS (ESI^+^): *m/z* 776.2089 (100%; M^+^ + 1H). IR (ATR): υ(CO) 1969 cm^–1^.

### Crystal Structure Determination of Complexes **2** and **5**

X-ray diffraction data were collected with an APEX DUO Bruker (compounds **2** and **5**) diffractometer, using graphite-monochromated Mo Kα radiation (λ = 0.71073 Å). Single crystals were mounted on a fiber and coated with a protecting perfluoropolyther oil. Data were collected at 100(2) K using ω-scans with a narrow oscillation frame strategy (Δω = 0.3°). Diffracted intensities were integrated and corrected of absorption effects by using a multiscan method using SAINT^32^ and SADABS^33^ programs integrated in the APEX2 package. Structures were solved by direct methods with SHELXS^34^ and refined by full-matrix least squares on F^2^ and SHELXL^35^ programs and the WINGx package.^36^

#### Structural Data for **2**

C_39_H_50_N_5_O_6_S_3_Rh; *M*_r_ = 883.93; yellow prism, 0.150 × 0.300 × 0.350 mm^3^; triclinic *P* – 1, *a* = 9.6962(8) Å, *b* = 14.3277(12) Å, *c* = 15.8986(13) Å, α = 90.5294(13)°, β = 93.6519(13)°, γ = 109.1142(13)°, *V* = 2081.6(3) Å^3^, *Z* = 2, *D*_c_ = 1.410 g/cm^3^; μ = 0.611 cm^–1^; min. and max. absorption correction factors: 0.846 and 0.914; 2θ_max_ = 56.502°; 23,223 reflections measured, 10,243 unique; *R*_int_ = 0.0297; number of data/restraint/parameters 10,243/0/ 537; *R*_1_ = 0.0382 [8629 reflections, *I* > 2σ(*I*)], w*R*(*F*^2^) = 0.0890 (all data); largest difference peak 0.833 e.Å^–3^.

#### Structural Data for **5**

C_40_H_38_N_5_ORh; *M*_r_ = 707.66; red prism, 0.090 × 0.230 × 0.240 mm^3^; monoclinic *P*2_1_/*c*, *a* = 10.7635(5) Å, *b* = 23.8996(11) Å, *c* = 13.7525(6) Å, β = 107.0640(10)°, *V* = 3382.0(3) Å^3^, *Z* = 4, *D*_c_ = 1.390 g/cm^3^; μ = 0.545 cm^–1^; min. and max. absorption correction factors: 0.8810 and 0.9281; 2θ_max_ = 60.398°; 40,194 reflections measured, 9374 unique; *R*_int_ = 0.0239; number of data/restraint/parameters 9374/1/450; *R*_1_ = 0.0347 [8014 reflections, *I* > 2*σ*(*I*)], *wR*(*F*^2^) = 0.0908 (all data); largest difference peak 1.197 e.Å^–3^.

CCDC 2142916 (**2**) and 1898012 (**5**) contain the supplementary crystallographic data for this paper. These data may be obtained free of charge from The Cambridge Crystallographic Data Centre via www.ccdc.cam.ac.uk/structures.

#### Computational Details

All DFT theoretical calculations were carried out using the Gaussian program package.^37^ The B3LYP-D3 method^38^ in combination to the def2-SVP basis set^39^ together with the corresponding core potential for Rh has been employed for geometry optimizations and vibrational frequencies. Energies were further refined by single point calculations using the M06L(SMD)/def2-TZVP level of theory^40,41^ including solvent corrections for toluene. The “ultrafine” grid was employed in all calculations. Relative energies are Gibbs free energies referred to a 1 M standard state using the approximation of Goddard et al.^42^ at 25 °C. The nature of the stationary points was confirmed by analytical frequency analysis, and transition states were characterized by a single imaginary frequency corresponding to the expected motion of the atoms.

## Supporting Information Available

NMR spectra (S2) and DFT studies (S18) (DOCX)
