# Supplementary material for: Nucleophilic Reactivity at a =CH Arm of a Lutidine-Based CNC/Rh System: Unusual Alkyne and CO2 Activation
Source: Inorg Chem. 2022 Apr 27;61(18):7120–9. doi: 10.1021/acs.inorgchem.2c00617 (PMC9994788; doi:10.1021/acs.inorgchem.2c00617)
Supplement: Supplementary file 1 — ic2c00617_si_001.docx [file ic2c00617_si_001.docx]

**Supporting Information**

Nucleophilic Reactivity at an =CH arm of a Lutidine-Based CNC/Rh System: Unusual Alkyne and CO_2_ Activation

Pablo Hermosilla,^a^ Pilar García-Orduña,^a^ Pablo J. Sanz Miguel,^a^ Víctor Polo,^b,^* Miguel A. Casado^a,^*

^a^ Instituto de Síntesis Química y Catálisis Homogénea (ISQCH), Departamento de Química Inorgánica, CSIC-Universidad de Zaragoza, c/ Pedro Cerbuna 12 50009, Zaragoza, Spain.

^b^ Departamento de Química Física and Instituto de Biocomputación y Física de los Sistemas Complejos (BIFI), Universidad de Zaragoza, c/ Pedro Cerbuna 12 50009, Zaragoza, Spain.

**Contents**

1. NMR spectra. S2
2. DFT calculations. S18
3. **NMR data**

**Figure S1.** ^1^H NMR spectrum of [(CNC*-*CO_2_)*^Mes^**Rh(CO)] (**2**) in DMSO-d_6_ at 298 K.

**

**

**Figure S2.** ^1^H-^1^H COSY NMR spectrum of [(CNC*-*CO_2_)*^Mes^**Rh(CO)] (**2**) in DMSO-d_6_ at 298 K.

**

**

**Figure S3.** ^13^C{^1^H}-APT NMR spectrum of [(CNC*-*CO_2_)*^Mes^**Rh(CO)] (**2**) in DMSO-d_6_ at 298 K.

**

**

**Figure S4.** ^1^H-^13^C HSQC NMR spectrum of [(CNC*-*CO_2_)*^Mes^**Rh(CO)] (**2**) in DMSO-d_6_ at 298 K.

**

**

**Figure S5.** Top: *In situ* ^1^H NMR spectrum of [(CNC)*^Mes^*Rh(CO)][HBzNC(O)O] (**3**) in DMSO-d_6_ at 298 K. The inset shows the presence of the anionic carbamoyl fragment and the formation of benzylcarbamic acid from carboxylation of benzylamine with free carbon dioxide in DMSO. Bottom: Complex **3** was obtained pure when CO_2_ is removed from the solvent under vacuum.

**

**

**

**

**Figure S6.** ^1^H-^1^H COSY NMR spectrum of [(CNC)*^Mes^*Rh(CO)][HBzNC(O)O] (**3**) in DMSO-d_6_ at 298 K.

**

**

**Figure S7.** ^13^C{^1^H}-APT NMR spectrum of [(CNC)*^Mes^*Rh(CO)][HBzNC(O)O] (**3**) in DMSO-d_6_ at 298 K.

**

**

**Figure S8.** ^1^H-^13^C HSQC NMR spectrum of [(CNC)*^Mes^*Rh(CO)][HBzNC(O)O] (**3**) in DMSO-d_6_ at 298 K.

**

**

**Figure S9.** ^1^H-^13^C HMBC NMR spectrum of [(CNC)*^Mes^*Rh(CO)][HBzNC(O)O] (**3**) in DMSO-d_6_ at 298 K.

**

**

**Figure S10.** ^1^H NMR spectrum of [(CNC)*^Mes^*Rh(CO)][H_2_NC(O)O] (**4**) in DMSO-d_6_ at 298 K.

**

**

**Figure S11.** ^1^H-^1^H COSY NMR spectrum of [(CNC)*^Mes^*Rh(CO)][H_2_NC(O)O] (**4**) in DMSO-d_6_ at 298 K.

**

**

**Figure S12.** ^13^C{^1^H}-APT NMR spectrum of [(CNC)*^Mes^**Rh(CO)][H_2_NC(O)O] (**4**) in DMSO-d_6_ at 298 K.

**

**

**Figure S13.** ^1^H-^13^C HSQC NMR spectrum of [(CNC)*^Mes^*Rh(CO)][H_2_NC(O)O] (**4**) in DMSO-d_6_ at 298 K.

**

**

**Figure S14.** Selected region of the ^1^H NMR spectrum of [(CNC*-*CH=CHPh)*^Mes^**Rh(CO)] (**5**) in C_6_D_6_ at 298 K.





**Figure S15.** ^1^H-^1^H COSY NMR spectrum of [(CNC*-*CH=CHPh)*^Mes^**Rh(CO)] (**5**) in C_6_D_6_ at 298 K.

**

**

**Figure S16.** Selected region of the ^13^C{^1^H}-APT NMR spectrum of [(CNC*-*CH=CHPh)*^Mes^**Rh(CO)] (**5**) in C_6_D_6_ at 298 K.

**

**

**Figure S17.** Selected region of the ^1^H-^13^C HSQC NMR spectrum of [(CNC*-*CH=CHPh)*^Mes^**Rh(CO)] (**5**) in C_6_D_6_ at 298 K.

**

**

**Figure S18.** ^1^H-^13^C HMBC NMR spectrum of [(CNC*-*CH=CHPh)*^Mes^**Rh(CO)] (**5**) in C_6_D_6_ at 298 K.





**Figure S19.** ^1^H NMR spectrum of [(CNC-CH=CH(2-py))*^Mes^**Rh(CO)] (**6**) in C_6_D_6_ at 298 K.





**Figure S20.** Selected region of the ^1^H-^1^H COSY NMR spectrum of [(CNC-CH=CH(2-py))*^Mes^**Rh(CO)] (**6**) in C_6_D_6_ at 298 K.





**Figure S21.** ^13^C{^1^H}-APT NMR spectrum of [(CNC-CH=CH(2-py))*^Mes^**Rh(CO)] (**6**) in C_6_D_6_ at 298 K.





**Figure S22.** Selected region of the ^1^H-^13^C HSQC NMR spectrum of [(CNC-CH=CH(2-py))*^Mes^**Rh(CO)] (**6**) in C_6_D_6_ at 298 K.





**Figure S23.** ^1^H-^13^C HMBC NMR spectrum of [(CNC-CH=CH(2-py))*^Mes^**Rh(CO)] (**6**) in C_6_D_6_ at 298 K.





**Figure S24.** ^1^H NMR spectrum of [(CNC-CH=CH(4-CF_3_-C_6_H_4_))*^Mes^**Rh(CO)] (**7**) in C_6_D_6_ at 298 K.

**

**

**Figure S25.** ^1^H-^1^H COSY NMR spectrum [(CNC-CH=CH(4-CF_3_-C_6_H_4_))*^Mes^**Rh(CO)] (**7**) in C_6_D_6_ at 298 K.





**Figure S26.** Selected region of ^13^C{^1^H}-APT NMR spectrum [(CNC-CH=CH(4-CF_3_-C_6_H_4_))*^Mes^**Rh(CO)] (**7**) in C_6_D_6_ at 298 K.





**Figure S27**: ^1^H-^13^C HSQC NMR spectrum [(CNC-CH=CH(4-CF_3_-C_6_H_4_))*^Mes^**Rh(CO)] (**7**) in C_6_D_6_ at 298 K.





**Figure S28.** ^1^H-^13^C HMBC NMR [(CNC-CH=CH(4-CF_3_-C_6_H_4_))*^Mes^**Rh(CO)] (**7**) in C_6_D_6_ at 298 K.





**Figure S29.** Deuterium labelling experiments with PhCCD: We show the olefinic area of the reaction of **1** with PhCCD in a 1:1.5 molar ratio, yielding [(CNC-CD=CHPh)*^Mes^**Rh(CO)] (**5-D**) (top, in blue) and with PhCCH (bottom, in red) yielding [(CNC-CH=CHPh)*^Mes^**Rh(CO)] (**5**) in C_6_D_6_ at 298 K.





**Figure S30.** Aliphatic region of the ^1^H NMR spectrum from the reaction of **1** with deuterated phenylacetylene in a 1:1.5 molar ratio, showing the formation of [(CNC-CH/D=CHPh)*^Mes^**Rh(CO)] (**5/5-D**) in C_6_D_6_ at 298 K.





1. **DFT Calculations**

**Table S1.** Energetic data for all DFT calculated structures. E(DZ) are the optimized energies at the B3LYP-D3/def2-SVP level. E(M06L,TZ,SMD) are the energy corrections at the M06L(SMD=toluene)/def2-TZVP level and Gcorr(1M) are the thermochemical corrections including the 1M standard state. DG are relative energies with respect to A and isolated molecules. Absolute energies in a.u., relative energies in kcalmol^−1^.

|  | E(DZ) | E(M06L,TZ,SMD) | Gcorr(1M) | DG |
| --- | --- | --- | --- | --- |
| **A** | -2005.59375 | -2007.2551 | 0.6084 | 0.0 |
| **a-TSAB** | -2005.54602 | -2007.2048 | 0.6099 | 32.5 |
| **a-B** | -2005.58123 | -2007.2346 | 0.6077 | 12.4 |
| **b-TSAB** | -2005.55993 | -2007.2174 | 0.6072 | 22.9 |
| **b-B** | -2005.59118 | -2007.2538 | 0.6120 | 3.1 |
| **c-TSAB** | -2005.56707 | -2007.2232 | 0.6076 | 19.5 |
| **c-B** | -2005.61543 | -2007.2701 | 0.6160 | -4.7 |
| **d-TSAB** | -2005.57309 | -2007.2335 | 0.6131 | 16.5 |
| **d-B** | -2005.57843 | -2007.2462 | 0.6108 | 7.1 |
| **d-TSBC_BH** | -2879.24772 | -2881.2946 | 0.8227 | 10.6 |
| **d-C_BH** | -2879.27744 | -2881.3275 | 0.8306 | -5.2 |
| **d-TSCD_BH** | -2879.27646 | -2881.3202 | 0.8282 | -2.1 |
| **D_BH** | -2879.30923 | -2881.3605 | 0.8329 | -24.4 |

**Figure S31.** Some geometrical parameters (left) and representation of the HOMO (right) of **d-B** intermediate.


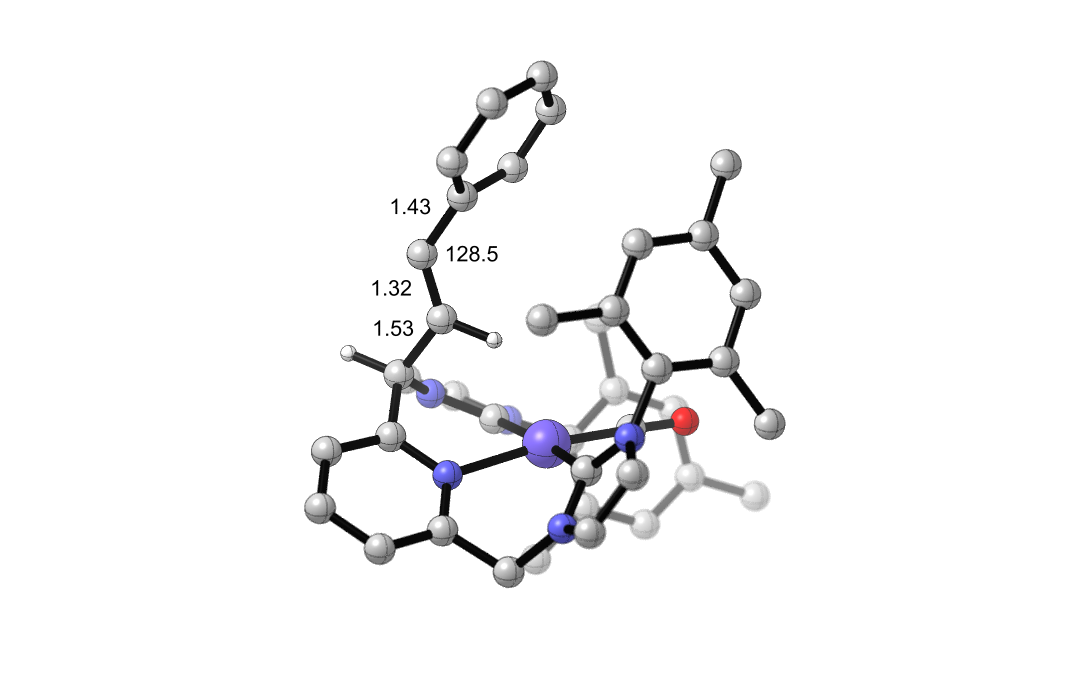

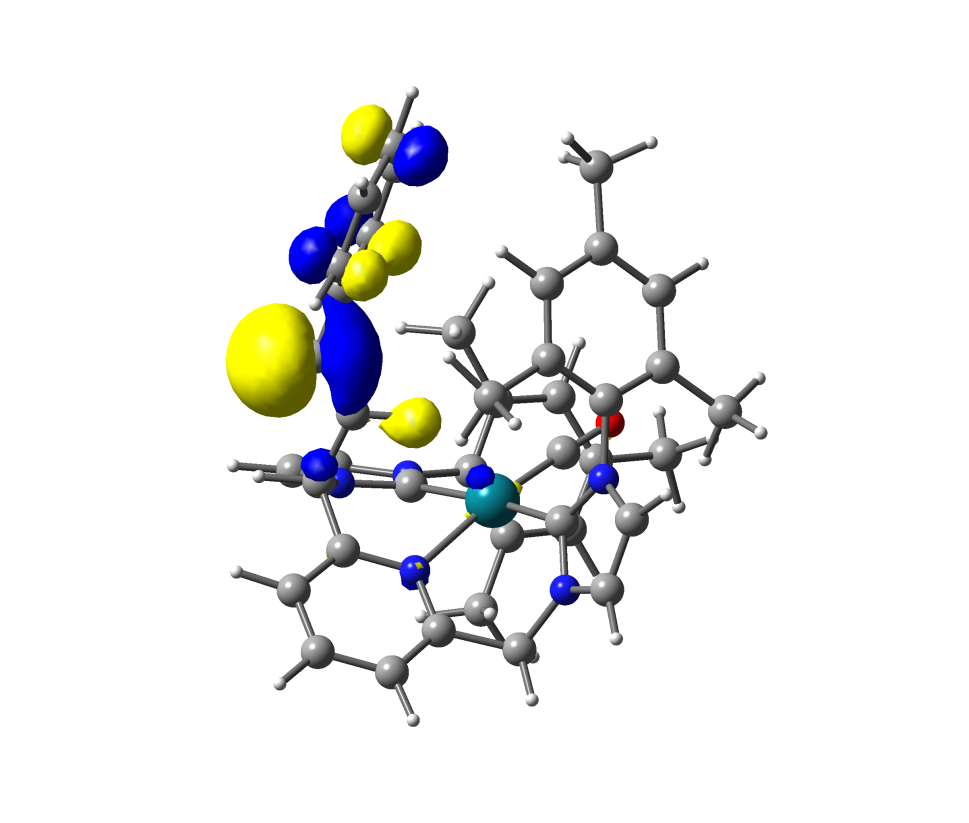


**Figure S32.** Geometrical representation of all DFT optimized structures.

**A** **a-TSAB**


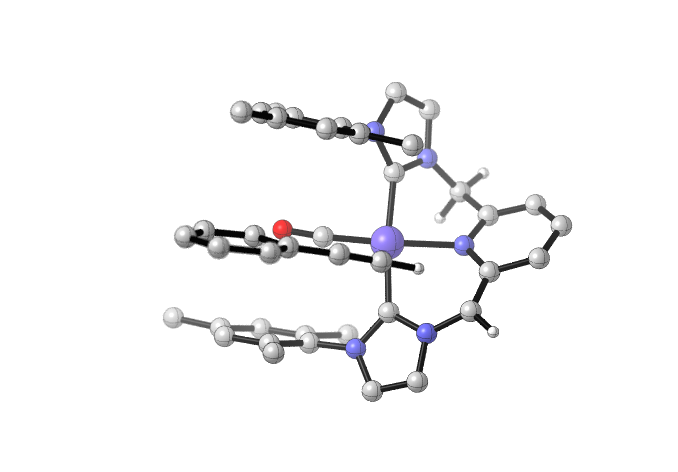

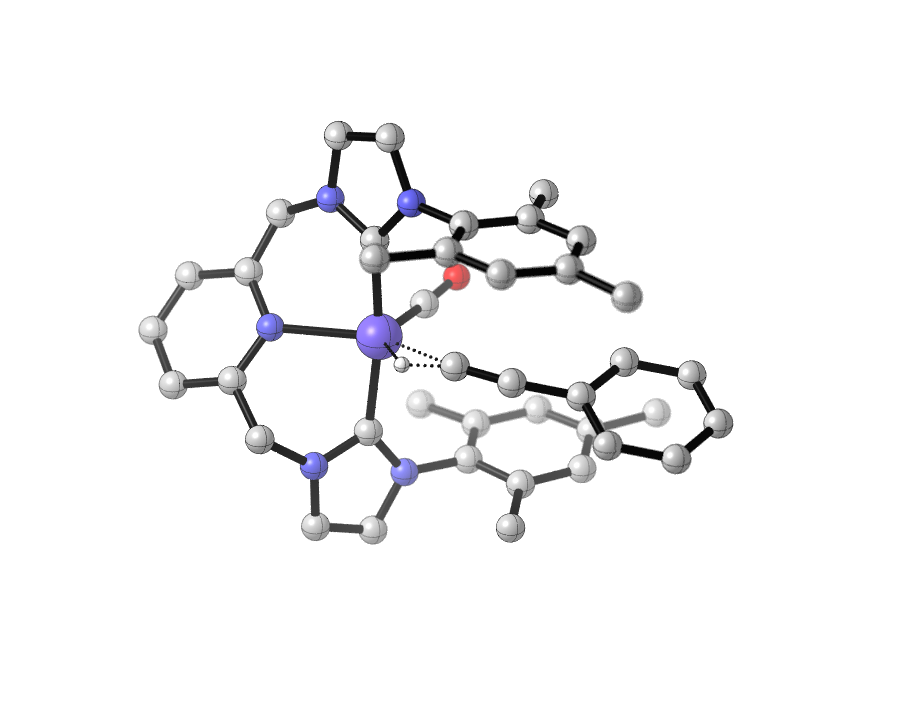


**a-B b-TSAB**


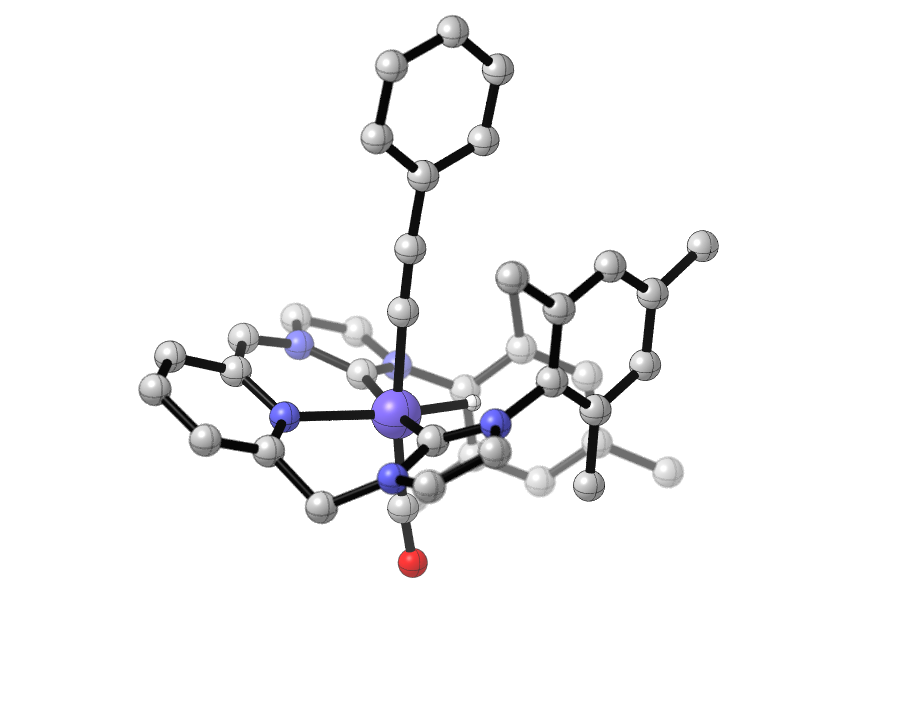

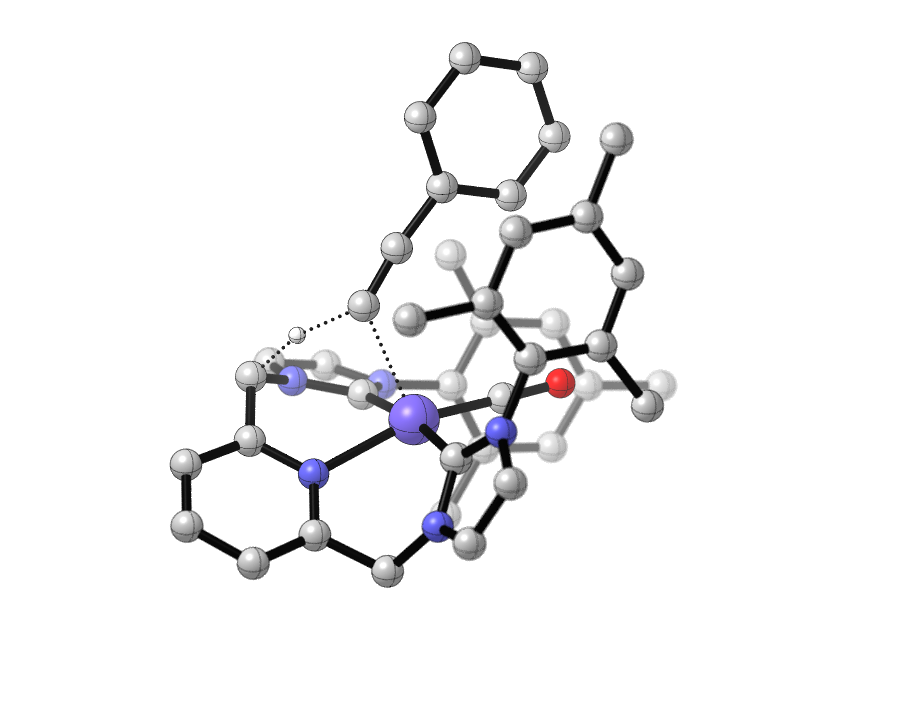


**b-B c-TSAB**


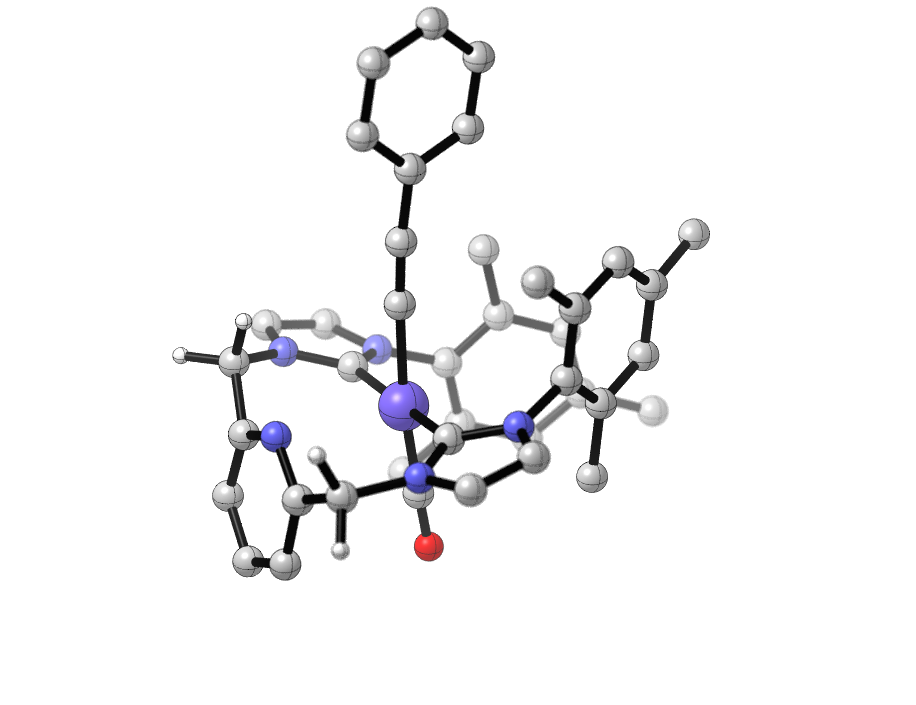

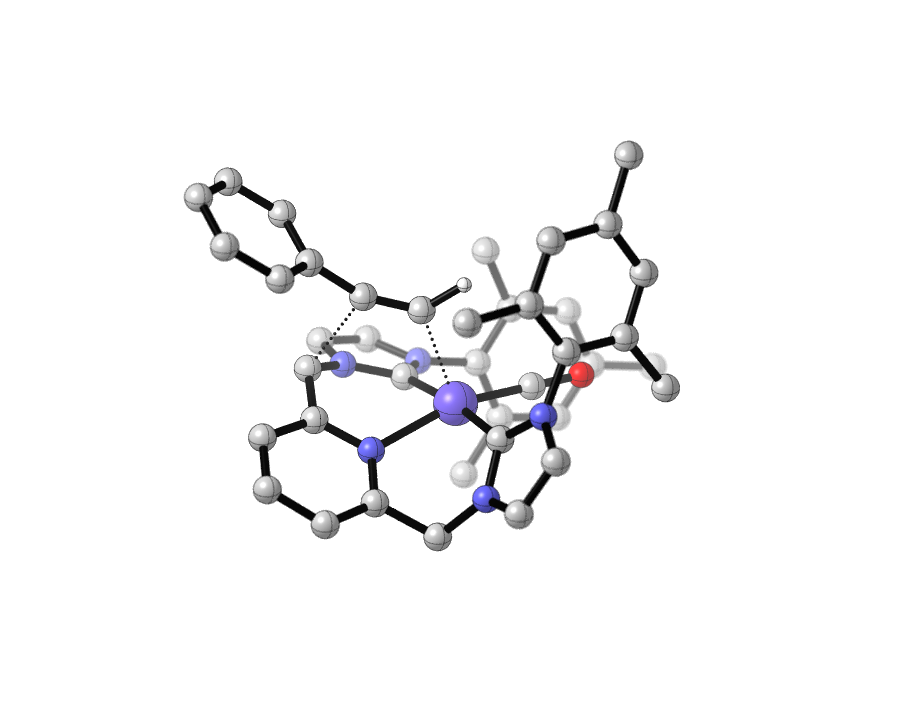


**c-B d-TSAB**


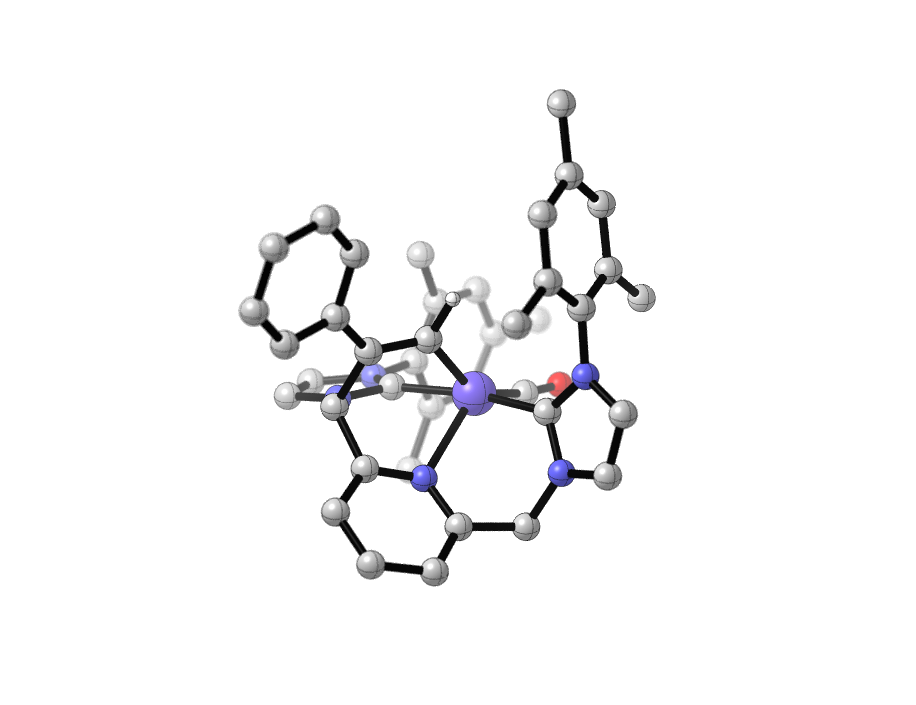

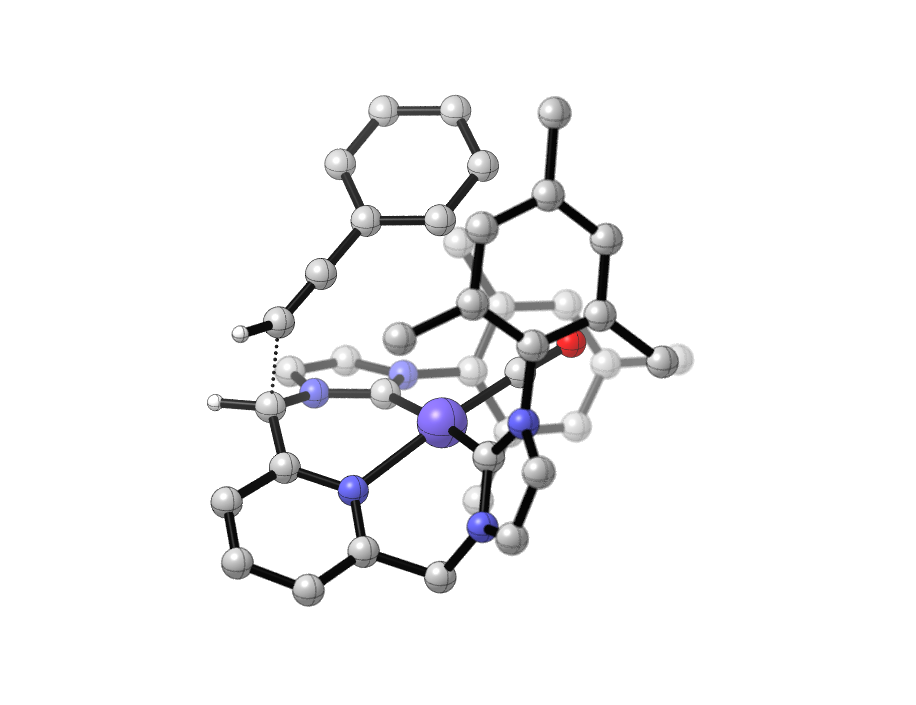


**d-B d-TSBC_BH**


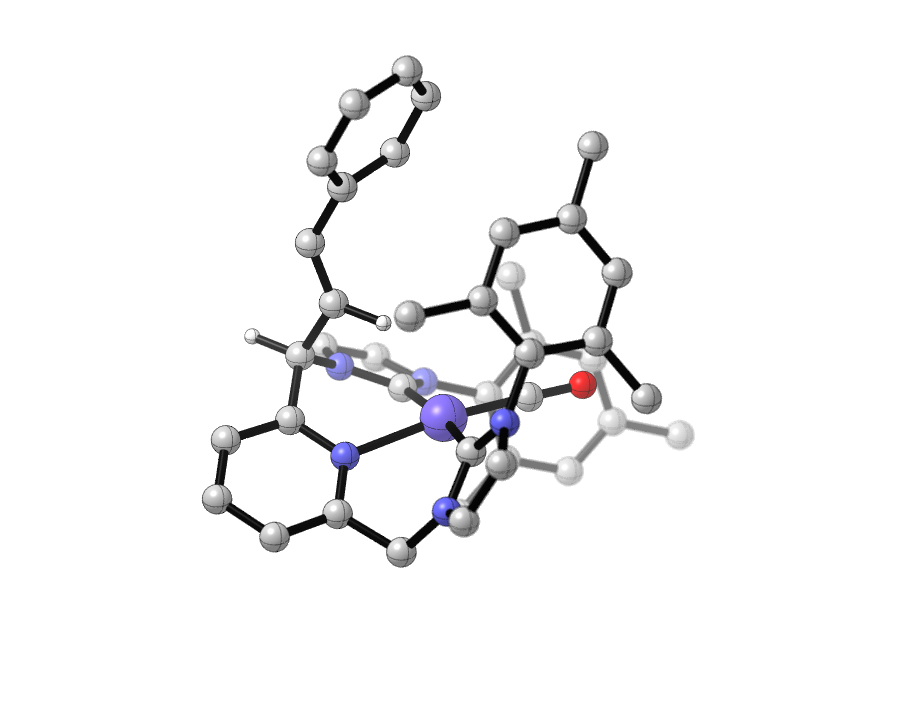

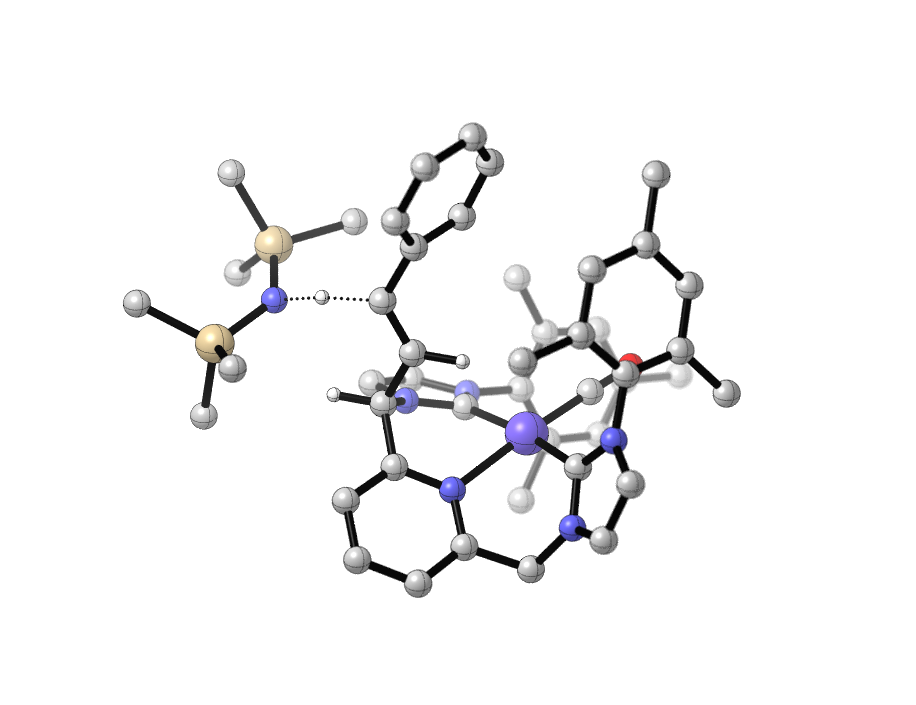


**d-C_BH d-TSCD_BH**


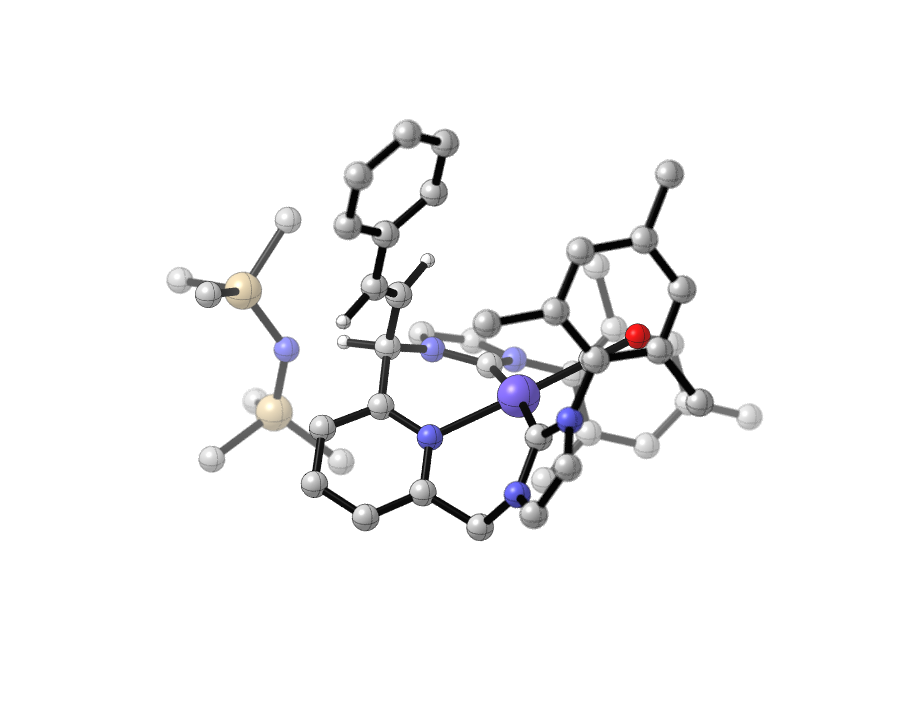

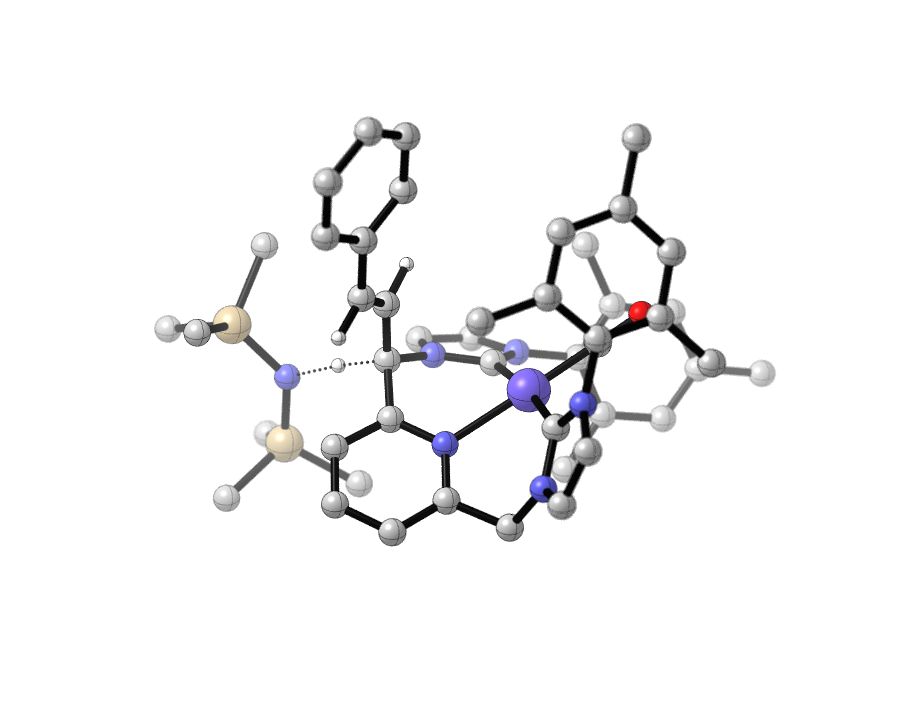


**D_BH**


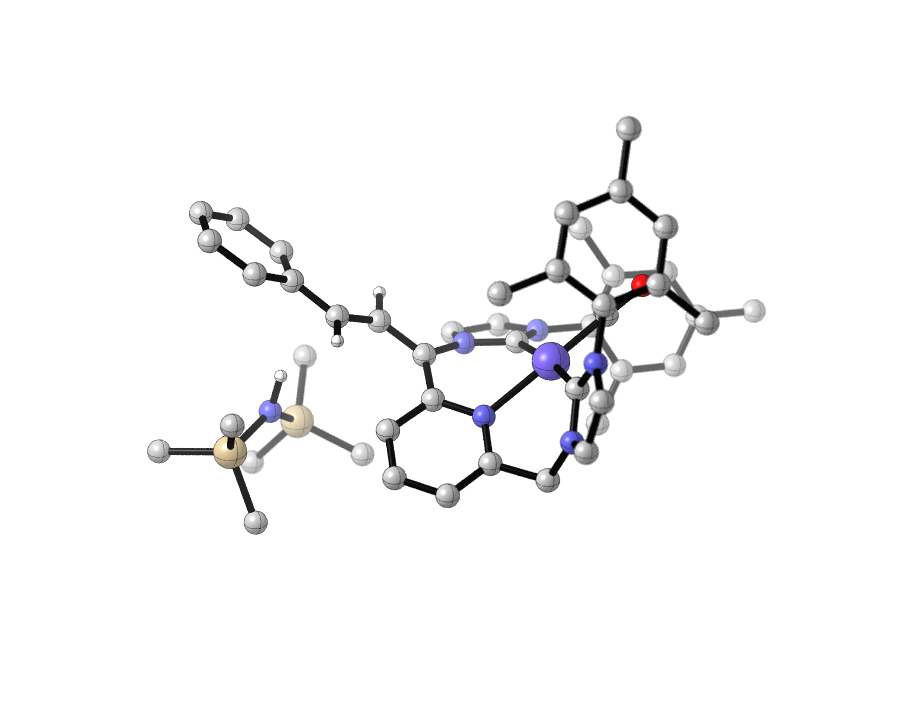


**Table S2.** Cartesian Coordinates for all DFT optimized species.

A

85

45 1.326373 0.387408 0.324847

7 1.291765 -2.520126 1.529457

7 3.151156 -1.476353 1.787838

7 3.279143 0.342925 -0.590081

7 1.555330 2.265631 -2.026542

7 0.044148 3.061785 -0.696727

6 1.890056 -1.311595 1.316182

6 2.181384 -3.420471 2.113612

1 1.891213 -4.439357 2.350387

6 3.360266 -2.759538 2.269023

6 4.159674 -0.449778 1.546440

6 4.330426 -0.229436 0.062061

6 5.486136 -0.651822 -0.557235

1 6.291291 -1.103219 0.023045

6 5.579765 -0.507580 -1.971629

1 6.455790 -0.882114 -2.507149

6 4.575245 0.122460 -2.643588

1 4.631636 0.281780 -3.722443

6 3.432758 0.663128 -1.935927

6 2.566453 1.490661 -2.623403

6 0.926473 2.014549 -0.842018

6 1.062576 3.429271 -2.613086

6 0.114415 3.926887 -1.775663

1 4.312060 -3.083200 2.679953

1 5.103193 -0.776204 2.000421

1 3.823763 0.479704 2.031282

1 1.450553 3.802963 -3.555264

1 -0.505697 4.814746 -1.843764

1 2.757782 1.731835 -3.666351

6 -0.285502 0.351664 1.167943

8 -1.273680 0.252685 1.766995

6 -0.373702 -0.335363 -2.790156

1 0.658428 -0.100609 -2.997876

6 -1.536192 -0.575172 -2.533040

6 -2.905810 -0.790680 -2.179848

6 -3.872068 -1.081401 -3.163079

6 -3.313302 -0.674917 -0.835712

6 -5.213511 -1.236669 -2.810792

1 -3.558839 -1.173132 -4.204836

6 -4.654916 -0.827879 -0.493304

1 -2.574550 -0.460883 -0.066694

6 -5.610584 -1.105456 -1.476278

1 -5.953854 -1.458264 -3.583299

1 -4.952675 -0.732810 0.553534

1 -6.661816 -1.225016 -1.203251

6 0.008822 -2.904804 1.011160

6 -0.088475 -3.190085 -0.363240

6 -1.086087 -3.017429 1.878986

6 -1.329807 -3.597923 -0.856339

6 -2.308490 -3.434274 1.335677

6 -2.448873 -3.731771 -0.024082

1 -1.427862 -3.805836 -1.924533

1 -3.176103 -3.518696 1.995454

6 -0.892570 3.196310 0.375165

6 -2.255989 2.974396 0.113255

6 -0.425029 3.524617 1.660070

6 -3.150495 3.053818 1.185985

6 -1.359467 3.591586 2.698125

6 -2.721519 3.346227 2.485279

1 -4.210851 2.862234 0.999741

1 -1.009304 3.834947 3.705198

6 -0.957024 -2.673093 3.339316

1 -1.904567 -2.848276 3.867454

1 -0.174110 -3.268135 3.835696

1 -0.688647 -1.612884 3.465220

6 1.104072 -3.042837 -1.267585

1 0.872695 -3.390046 -2.282905

1 1.410686 -1.986508 -1.323891

1 1.971168 -3.608329 -0.889854

6 1.038722 3.756181 1.916174

1 1.586673 2.799096 1.865239

1 1.483861 4.412859 1.152917

1 1.199596 4.205291 2.906444

6 -2.744208 2.650938 -1.274375

1 -2.066085 1.955749 -1.787220

1 -3.742903 2.196266 -1.241328

1 -2.808229 3.557834 -1.898944

6 -3.764979 -4.189391 -0.595613

1 -3.724542 -5.257448 -0.870135

1 -4.586446 -4.055995 0.122302

1 -4.016550 -3.624588 -1.505573

6 -3.694503 3.356290 3.636280

1 -3.395756 4.076169 4.413533

1 -4.712937 3.609138 3.304855

1 -3.741049 2.361357 4.112492

a-TSAB

85

45 -1.272796 -0.140132 0.063967

7 -0.793607 2.916989 0.798024

7 -2.293762 1.936979 1.981346

7 -3.499373 -0.324754 0.423050

7 -2.558586 -2.425277 -1.407936

7 -0.574133 -3.088728 -0.844023

6 -1.406577 1.710864 0.979711

6 -1.311978 3.876271 1.660968

1 -0.952814 4.900497 1.658834

6 -2.265669 3.255664 2.405607

6 -3.187275 0.894066 2.484319

6 -4.098020 0.398312 1.393194

6 -5.433681 0.757891 1.391231

1 -5.852466 1.354900 2.201802

6 -6.218081 0.352436 0.277254

1 -7.270827 0.640030 0.211915

6 -5.650665 -0.410086 -0.704199

1 -6.237522 -0.755476 -1.557979

6 -4.267348 -0.826047 -0.616396

6 -3.768732 -1.711126 -1.554225

6 -1.435602 -2.023873 -0.754978

6 -2.398496 -3.715023 -1.904835

6 -1.151632 -4.129779 -1.554154

1 -2.918215 3.629374 3.188891

1 -3.763047 1.316343 3.316674

1 -2.563606 0.066069 2.857749

1 -3.194977 -4.218942 -2.442773

1 -0.623540 -5.061532 -1.730328

1 -4.410624 -2.089423 -2.345932

6 -0.890285 0.431576 -1.709712

8 -0.523795 0.550366 -2.796036

1 -0.278453 -0.802760 1.494398

6 0.618713 -0.352448 0.865821

6 1.841161 -0.196644 0.950406

6 3.228328 0.067781 0.792574

6 3.782889 0.177993 -0.503896

6 4.084022 0.252465 1.899538

6 5.136302 0.453046 -0.679769

1 3.125632 0.052815 -1.364759

6 5.437790 0.538977 1.716569

1 3.668322 0.166990 2.905258

6 5.972549 0.638780 0.428271

1 5.544899 0.530077 -1.690839

1 6.082727 0.679960 2.587700

1 7.033478 0.858173 0.287643

6 0.726024 -3.196377 -0.253248

6 0.825549 -3.627811 1.076978

6 1.856824 -2.896491 -1.029154

6 2.103877 -3.732149 1.637938

6 3.111255 -3.034878 -0.432594

6 3.255438 -3.433549 0.902724

1 2.199003 -4.047230 2.680631

1 4.002696 -2.788234 -1.014017

6 0.306146 3.159612 -0.091232

6 1.607250 3.136583 0.443796

6 0.055371 3.422282 -1.444180

6 2.676227 3.305128 -0.437795

6 1.162150 3.574836 -2.290993

6 2.473529 3.501225 -1.811462

1 3.694709 3.257867 -0.045320

1 0.987894 3.747091 -3.356201

6 1.712230 -2.376323 -2.433599

1 1.062209 -3.017601 -3.048480

6 -0.412690 -3.930259 1.878547

1 -1.032825 -3.027074 2.002190

6 1.835474 2.959153 1.920931

1 1.456964 3.824361 2.490092

6 -1.349464 3.578167 -1.966994

1 -2.042029 2.852713 -1.519265

1 2.905701 2.850190 2.136817

1 -1.737631 4.585171 -1.735207

1 -1.377935 3.448885 -3.057303

1 1.318504 2.064315 2.295871

1 -0.154127 -4.310914 2.876403

1 1.252117 -1.375447 -2.430437

1 2.690996 -2.301424 -2.927479

1 -1.047768 -4.677294 1.376077

6 4.622591 -3.480804 1.532497

1 4.995619 -2.456808 1.703946

1 4.607534 -4.002037 2.500947

1 5.351034 -3.986658 0.879226

6 3.656666 3.621135 -2.736882

1 4.273071 2.707812 -2.697591

1 3.344432 3.778809 -3.779263

1 4.310276 4.459500 -2.444807

a-B

85

45 -0.256424 -1.186118 0.300896

7 -0.552642 0.725623 2.757736

7 -1.752466 -1.055831 2.898269

7 -2.207731 -2.242618 0.109278

7 -0.687124 -2.119746 -2.536457

7 1.401297 -1.583333 -2.275497

6 -0.846702 -0.434878 2.112860

6 -1.281585 0.825334 3.939073

1 -1.188423 1.681329 4.599412

6 -2.041638 -0.300521 4.026813

6 -2.306435 -2.367540 2.560301

6 -2.980089 -2.431082 1.206222

6 -4.329035 -2.724536 1.164836

1 -4.896886 -2.868025 2.084323

6 -4.942048 -2.838731 -0.114639

1 -6.011421 -3.050197 -0.195672

6 -4.182154 -2.691560 -1.235993

1 -4.626416 -2.786902 -2.228825

6 -2.760374 -2.412085 -1.153884

6 -2.064460 -2.349023 -2.347264

6 0.207561 -1.697585 -1.615104

6 -0.059055 -2.268034 -3.772743

6 1.251545 -1.929878 -3.607689

1 -2.749359 -0.627567 4.782686

1 -3.028174 -2.630363 3.342182

1 -1.483721 -3.100170 2.597523

1 -0.603983 -2.601167 -4.650121

1 2.080960 -1.905438 -4.307167

6 0.767657 -2.592437 1.089740

8 1.459552 -3.362255 1.582982

6 2.620308 -1.177565 -1.642367

6 2.915020 0.193431 -1.540213

6 3.448220 -2.162714 -1.081030

6 4.059983 0.561119 -0.825941

6 4.572745 -1.743288 -0.359048

6 4.885825 -0.386946 -0.209011

1 4.300068 1.623171 -0.735702

1 5.215741 -2.498121 0.101722

6 0.319325 1.742443 2.238845

6 -0.265104 2.907419 1.704633

6 1.705726 1.531494 2.231613

6 0.580283 3.849523 1.115327

6 2.509425 2.510611 1.630896

6 1.967897 3.659586 1.050512

1 0.136752 4.744594 0.672763

1 3.591117 2.357268 1.612429

6 2.004361 1.231815 -2.131415

1 1.719872 0.986313 -3.165748

6 3.142091 -3.626966 -1.260283

1 2.068708 -3.836997 -1.155869

6 -1.752017 3.136707 1.759330

6 2.352115 0.298427 2.806777

1 1.677062 -0.260260 3.467923

1 -2.605446 -2.488669 -3.279431

1 1.017374 -0.315197 0.356044

1 2.483841 2.220725 -2.124860

1 1.067382 1.293820 -1.554996

1 3.434972 -3.967934 -2.267856

1 -2.044428 3.940734 1.071965

1 2.664099 -0.382187 1.998717

1 -2.077842 3.420970 2.774235

1 3.686419 -4.236597 -0.526074

1 3.253403 0.567947 3.378363

6 -1.082372 0.443036 -0.536899

1 -2.301616 2.231247 1.469169

6 -1.522669 1.461293 -1.057430

6 -1.993856 2.694364 -1.596531

6 -3.374463 2.955560 -1.735201

6 -1.083619 3.708234 -1.970996

6 -3.823071 4.183905 -2.219218

1 -4.085349 2.177327 -1.450751

6 -1.538768 4.934263 -2.454652

1 -0.014851 3.516844 -1.867015

6 -2.910124 5.181169 -2.579328

1 -4.896521 4.366496 -2.316491

1 -0.817092 5.705378 -2.737590

1 -3.265069 6.142929 -2.957429

6 6.060177 0.051889 0.628051

1 5.732858 0.311629 1.650324

1 6.816198 -0.742066 0.718363

1 6.546208 0.945035 0.206347

6 2.843446 4.658091 0.338032

1 2.550505 5.693774 0.570205

1 3.903466 4.531904 0.603703

1 2.758493 4.537032 -0.756141

b-TSAB

85

45 -1.160953 -0.069465 -0.200274

7 -0.075783 -2.612692 -1.760404

7 -2.183335 -2.253456 -1.951942

7 -3.077702 -0.865737 0.534983

7 -2.222108 1.354627 2.167725

7 -1.320072 2.876564 0.933695

6 -1.056653 -1.742703 -1.381424

6 -0.589296 -3.641400 -2.545482

1 0.045310 -4.429327 -2.938841

6 -1.925484 -3.417935 -2.660273

6 -3.482851 -1.635722 -1.731455

6 -3.840758 -1.645602 -0.269056

6 -4.876466 -2.432322 0.215024

1 -5.464290 -3.043345 -0.471785

6 -5.147753 -2.421361 1.593508

1 -5.952403 -3.034472 2.005745

6 -4.396761 -1.598512 2.413413

1 -4.594122 -1.537941 3.485217

6 -3.377688 -0.790998 1.861994

6 -2.536549 0.048799 2.692903

6 -1.569823 1.533349 0.987833

6 -2.373136 2.557105 2.844732

6 -1.799890 3.517802 2.069406

1 -2.701065 -3.970174 -3.182736

1 -4.230937 -2.179173 -2.322422

1 -3.418221 -0.588516 -2.073403

1 -2.875704 2.621042 3.804999

1 -1.691745 4.588254 2.213324

1 -2.843874 0.113278 3.739930

6 0.157815 0.735416 -1.179394

8 1.039752 1.157833 -1.802695

6 -0.076927 -0.739536 1.831788

1 -1.321082 -0.540359 2.460837

6 1.135172 -0.618310 2.053560

6 2.539168 -0.388670 2.146693

6 3.298674 -0.835076 3.249564

6 3.205969 0.323258 1.122665

6 4.663697 -0.558457 3.336804

1 2.795777 -1.393416 4.041827

6 4.568273 0.596956 1.217432

1 2.639288 0.649950 0.250779

6 5.304949 0.165703 2.326601

1 5.231616 -0.906962 4.203578

1 5.062669 1.148228 0.413125

1 6.373343 0.383605 2.398085

6 -0.514803 3.502406 -0.069945

6 0.841856 3.729375 0.216295

6 -1.084687 3.820505 -1.311370

6 1.630568 4.303292 -0.785134

6 -0.253962 4.390040 -2.282205

6 1.103484 4.633333 -2.039978

1 2.692119 4.474162 -0.586186

1 -0.677764 4.637493 -3.259476

6 1.289123 -2.576502 -1.316130

6 2.263428 -1.964885 -2.115251

6 1.601464 -3.211186 -0.099371

6 3.586448 -1.977565 -1.650849

6 2.934143 -3.203457 0.313589

6 3.940218 -2.590777 -0.446260

1 4.358892 -1.489227 -2.251360

1 3.193220 -3.671699 1.266376

6 1.425711 3.319666 1.542629

1 1.067173 3.965172 2.361457

1 1.138167 2.288758 1.799145

1 2.522263 3.370980 1.523794

6 -2.526176 3.498427 -1.594722

1 -2.661903 2.403606 -1.629524

1 -3.190682 3.876562 -0.802063

1 -2.847273 3.925569 -2.555189

6 0.524600 -3.863139 0.723107

1 -0.175245 -3.097926 1.094169

1 -0.049847 -4.595997 0.133594

1 0.955174 -4.377582 1.592990

6 1.927109 -1.352721 -3.450314

1 0.885777 -1.011427 -3.498063

1 2.573697 -0.488365 -3.655624

1 2.076515 -2.085234 -4.262936

6 5.368618 -2.613699 0.030968

1 5.427421 -2.380076 1.103534

1 5.817822 -3.610682 -0.120454

1 5.985880 -1.880867 -0.508099

6 1.992364 5.188215 -3.123504

1 1.423246 5.792758 -3.845678

1 2.796669 5.813834 -2.707374

1 2.472857 4.368942 -3.686141

b-B

85

45 0.924257 -0.532700 0.165482

7 1.408792 1.211926 -2.344737

7 1.889763 -0.855790 -2.692517

7 2.076546 -3.008924 -0.154210

7 0.747583 -2.375985 2.657992

7 -0.654164 -0.744124 2.778624

6 1.438582 -0.002175 -1.730878

6 1.824372 1.120565 -3.670433

1 1.863524 1.985909 -4.324656

6 2.124648 -0.188415 -3.889211

6 2.072004 -2.294824 -2.467094

6 2.812737 -2.543587 -1.166875

6 4.151923 -2.168326 -1.003231

1 4.728420 -1.798264 -1.853944

6 4.713930 -2.234384 0.271510

1 5.751248 -1.936589 0.438260

6 3.917475 -2.655612 1.338872

1 4.307439 -2.669679 2.358483

6 2.598147 -3.037574 1.078410

6 1.648898 -3.442391 2.178767

6 0.311305 -1.278143 1.974334

6 0.061998 -2.519796 3.863042

6 -0.820810 -1.491322 3.938343

1 2.471872 -0.698821 -4.782896

1 2.625019 -2.692349 -3.330231

1 1.086869 -2.776625 -2.402741

1 0.265246 -3.335113 4.551237

1 -1.540237 -1.217000 4.703453

1 1.022931 -4.267290 1.805199

6 2.385293 0.329108 0.932420

8 3.277702 0.887338 1.418120

6 -0.808224 -1.242371 -0.654719

6 -1.883982 -1.589039 -1.146000

6 -3.188018 -1.894197 -1.633220

6 -3.567475 -3.210934 -1.977422

6 -4.159036 -0.872853 -1.760580

6 -4.858454 -3.491299 -2.424207

1 -2.829621 -4.010242 -1.881962

6 -5.448100 -1.160197 -2.206455

1 -3.879248 0.147843 -1.495098

6 -5.807829 -2.470481 -2.541044

1 -5.128624 -4.518797 -2.682846

1 -6.181159 -0.353540 -2.294894

1 -6.818856 -2.693842 -2.890229

6 -1.518727 0.330413 2.372719

6 -2.803504 -0.011307 1.902350

6 -1.075204 1.658450 2.421997

6 -3.629003 1.018053 1.446061

6 -1.950364 2.655630 1.968376

6 -3.215717 2.357786 1.460463

1 -4.617651 0.762028 1.056271

1 -1.616591 3.694959 2.003899

6 1.094509 2.425470 -1.645130

6 -0.247608 2.792544 -1.465919

6 2.156726 3.180221 -1.117511

6 -0.508056 3.951968 -0.725230

6 1.843158 4.317060 -0.366280

6 0.515838 4.712790 -0.152298

1 -1.547268 4.253074 -0.578131

1 2.657232 4.903547 0.068495

6 0.294486 2.051094 2.904296

1 0.809508 1.232593 3.421867

6 -3.274782 -1.440956 1.881143

1 -2.509302 -2.097783 1.446545

6 -1.369661 1.946892 -1.994200

1 -1.480800 1.040683 -1.375859

6 3.587254 2.768000 -1.347662

1 3.736563 1.700069 -1.134648

1 2.212106 -3.799917 3.052763

1 0.231007 2.913258 3.586815

1 0.922372 2.347884 2.050308

1 -3.502092 -1.806804 2.896649

1 -4.182920 -1.539328 1.272137

1 -2.318536 2.501366 -1.981147

1 -1.179153 1.600176 -3.020940

1 3.888700 2.933917 -2.395620

1 4.268132 3.340904 -0.703559

6 0.198185 5.904154 0.714838

1 0.212141 5.622496 1.782622

1 0.934835 6.711803 0.584693

1 -0.800708 6.309602 0.495433

6 -4.105425 3.440229 0.904877

1 -5.152839 3.306746 1.217734

1 -3.775992 4.439949 1.224608

1 -4.095327 3.424779 -0.199094

c-TSAB

85

45 -0.261190 -0.147846 -0.835095

7 0.037082 2.992363 -1.277996

7 0.478327 1.788006 -2.998502

7 1.459823 -0.845290 -1.957910

7 0.708781 -2.802622 0.053703

7 -1.435959 -2.809640 0.330297

6 0.065250 1.692922 -1.706406

6 0.432215 3.865976 -2.287545

1 0.471041 4.939713 -2.132578

6 0.718684 3.100443 -3.374403

6 0.742285 0.587716 -3.782963

6 1.812833 -0.236418 -3.123226

6 3.082377 -0.311384 -3.666292

1 3.304430 0.196855 -4.605950

6 4.071325 -1.058161 -2.985682

1 5.088062 -1.122622 -3.379612

6 3.717377 -1.730684 -1.838868

1 4.436873 -2.349612 -1.300373

6 2.380749 -1.666282 -1.349569

6 2.030082 -2.380055 -0.185277

6 -0.405114 -2.039741 -0.146145

6 0.382250 -4.010202 0.666221

6 -0.966228 -4.013305 0.838545

1 1.057681 3.371060 -4.370021

1 1.042920 0.893939 -4.792756

1 -0.193235 0.003311 -3.824178

1 1.135437 -4.756200 0.899452

1 -1.634671 -4.758113 1.258982

1 2.774489 -3.052053 0.235937

6 -1.949020 0.391467 -0.336756

8 -2.965718 0.849769 -0.019909

6 2.035270 -0.611161 1.256255

6 1.016844 0.108073 1.032036

1 0.578893 0.952239 1.559092

6 3.284946 -0.936531 1.871352

6 3.361253 -1.821119 2.971890

6 4.490449 -0.402056 1.360160

6 4.591395 -2.149002 3.537696

1 2.436653 -2.243967 3.371153

6 5.717722 -0.740527 1.930366

1 4.443743 0.277710 0.507566

6 5.778290 -1.614481 3.020711

1 4.627645 -2.829429 4.392833

1 6.637675 -0.314992 1.520349

1 6.740960 -1.876071 3.466152

6 -0.193384 3.394447 0.078157

6 0.910574 3.408786 0.953579

6 -1.479444 3.767934 0.489079

6 0.681323 3.757841 2.286087

6 -1.658457 4.106269 1.838655

6 -0.599603 4.095035 2.750649

1 1.524049 3.759036 2.982803

1 -2.659320 4.382279 2.181686

6 -2.801329 -2.393166 0.401248

6 -3.285069 -1.890205 1.619843

6 -3.603441 -2.472227 -0.747473

6 -4.619601 -1.478112 1.673439

6 -4.929912 -2.039796 -0.647418

6 -5.454470 -1.539994 0.550860

1 -5.009782 -1.072435 2.610958

1 -5.568015 -2.086994 -1.534289

6 -2.629381 3.857466 -0.479390

1 -3.572468 3.574246 0.007107

1 -2.736915 4.891457 -0.851432

1 -2.489522 3.202520 -1.347580

6 2.289400 3.064395 0.455237

1 2.643152 3.809016 -0.277041

1 3.008374 3.027084 1.284632

1 2.295694 2.082976 -0.040606

6 -3.025135 -2.955547 -2.048749

1 -2.275891 -2.233088 -2.415502

1 -2.504399 -3.918498 -1.928945

1 -3.807019 -3.072028 -2.812293

6 -2.364818 -1.741704 2.802889

1 -2.027197 -2.717710 3.187685

1 -1.457377 -1.184125 2.519852

1 -2.863072 -1.206453 3.623029

6 -0.818417 4.422702 4.205850

1 -0.726046 3.517622 4.829968

1 -0.069721 5.143236 4.571928

1 -1.817519 4.847867 4.380046

6 -6.870825 -1.028450 0.617648

1 -6.903207 0.051863 0.392811

1 -7.519896 -1.534744 -0.112686

1 -7.305076 -1.166705 1.619482

c-B

85

45 0.575600 -0.147550 0.619626

7 -1.270674 1.910303 2.181706

7 -0.654392 0.197261 3.326404

7 -0.545259 -2.103535 1.483470

7 0.453206 -2.446745 -1.211437

7 2.432931 -1.627309 -1.422489

6 -0.475364 0.801295 2.111458

6 -1.926901 1.990768 3.408008

1 -2.589093 2.815760 3.651514

6 -1.544433 0.903768 4.126822

6 -0.113688 -1.125784 3.631975

6 -0.794061 -2.187171 2.793119

6 -1.670897 -3.146395 3.306866

1 -1.870897 -3.201073 4.379009

6 -2.280857 -4.032429 2.412468

1 -2.957437 -4.807554 2.779830

6 -2.032979 -3.905807 1.044139

1 -2.513625 -4.571595 0.324470

6 -1.164660 -2.897574 0.608450

6 -0.968110 -2.552674 -0.862411

6 1.245301 -1.422025 -0.781910

6 1.129342 -3.274764 -2.097021

6 2.380048 -2.758323 -2.234653

1 -1.807390 0.584474 5.131057

1 -0.244372 -1.314357 4.705644

1 0.958236 -1.111121 3.375960

1 0.662744 -4.140947 -2.556943

1 3.226091 -3.073594 -2.837360

1 -1.375010 -3.364464 -1.475434

6 2.190218 0.656527 1.078403

8 3.100323 1.352529 1.296996

6 -1.672757 -1.218708 -1.159459

6 -1.104959 -0.110510 -0.626453

1 -1.594660 0.840893 -0.848680

6 -2.930490 -1.239813 -1.937135

6 -3.751630 -2.382708 -2.028873

6 -3.384038 -0.074388 -2.596986

6 -4.960864 -2.363498 -2.729343

1 -3.462672 -3.304455 -1.520961

6 -4.589721 -0.053338 -3.292788

1 -2.765837 0.824150 -2.564928

6 -5.392026 -1.199077 -3.366216

1 -5.573107 -3.268558 -2.770045

1 -4.903744 0.865550 -3.795764

1 -6.336046 -1.182762 -3.915863

6 -1.554882 2.773843 1.071891

6 -2.778707 2.595251 0.395992

6 -0.618839 3.738324 0.676178

6 -3.043644 3.412381 -0.705810

6 -0.932444 4.532612 -0.436625

6 -2.130501 4.383735 -1.140806

1 -3.981398 3.271591 -1.250524

1 -0.210811 5.287578 -0.760445

6 3.566497 -0.763652 -1.291928

6 3.563284 0.465248 -1.970564

6 4.618823 -1.151713 -0.449009

6 4.669076 1.304146 -1.799421

6 5.704279 -0.281395 -0.313154

6 5.744508 0.951423 -0.975586

1 4.681262 2.269442 -2.313150

1 6.528159 -0.563114 0.348388

6 0.692464 3.908724 1.391924

1 1.438054 3.193160 1.014420

1 1.088758 4.923832 1.244686

1 0.596494 3.722952 2.471445

6 -3.754584 1.526006 0.815653

1 -4.265202 1.783410 1.758075

1 -4.519683 1.375212 0.042259

1 -3.240764 0.565711 0.971735

6 4.534849 -2.434797 0.334601

1 3.630401 -2.434439 0.965055

1 4.467699 -3.318731 -0.319767

1 5.411892 -2.557028 0.985338

6 2.376176 0.887113 -2.792294

1 2.048978 0.090516 -3.478390

1 1.522721 1.101642 -2.126229

1 2.602586 1.786633 -3.381771

6 -2.442737 5.235634 -2.345138

1 -3.378000 5.801924 -2.202771

1 -1.638098 5.956229 -2.551012

1 -2.577768 4.613611 -3.245589

6 6.892345 1.904070 -0.759798

1 6.675956 2.582410 0.083725

1 7.824346 1.369904 -0.520563

1 7.071373 2.530961 -1.646556

d-TSAB

85

45 0.740870 -0.662906 -0.438673

7 2.246698 1.857301 -1.579742

7 3.206150 -0.019525 -1.989627

7 2.452172 -1.751221 0.328441

7 0.055168 -2.452494 1.863443

7 -1.662001 -2.421868 0.556189

6 2.090106 0.511163 -1.425811

6 3.446765 2.151782 -2.222674

1 3.749983 3.173611 -2.428684

6 4.057770 0.962386 -2.474975

6 3.480353 -1.445629 -1.867994

6 3.584729 -1.843938 -0.413251

6 4.804393 -2.241794 0.107446

1 5.683270 -2.297509 -0.535797

6 4.879998 -2.555326 1.483465

1 5.831249 -2.846438 1.934784

6 3.728825 -2.519059 2.234386

1 3.740323 -2.794769 3.290744

6 2.483230 -2.169944 1.638363

6 1.310988 -2.163050 2.459831

6 -0.401526 -1.903829 0.706352

6 -0.917846 -3.245998 2.459476

6 -1.994681 -3.236179 1.630407

1 5.002883 0.733092 -2.958541

1 4.415404 -1.668234 -2.396451

1 2.653742 -1.995416 -2.343960

1 -0.749204 -3.752425 3.404591

1 -2.955754 -3.735019 1.698555

1 1.431594 -2.724678 3.387582

6 -0.673751 0.198037 -1.208794

8 -1.524695 0.765704 -1.746724

6 1.115584 -0.348185 3.193181

1 2.074926 -0.213536 3.691609

6 0.048718 0.287797 3.000715

6 -1.181116 0.931686 2.801392

6 -2.209646 0.873177 3.785989

6 -1.446352 1.702492 1.635029

6 -3.408064 1.559888 3.613453

1 -2.038715 0.284585 4.689803

6 -2.647603 2.382150 1.478295

1 -0.689143 1.742302 0.857682

6 -3.642844 2.326830 2.464387

1 -4.175099 1.495674 4.390621

1 -2.811614 2.950184 0.559071

1 -4.588090 2.858399 2.333757

6 1.405002 2.844499 -0.963429

6 1.609943 3.126526 0.399290

6 0.424690 3.493591 -1.726268

6 0.786082 4.084085 0.995323

6 -0.383337 4.437506 -1.079017

6 -0.221694 4.741577 0.277645

1 0.913272 4.297881 2.059398

1 -1.165568 4.941555 -1.652725

6 -2.521088 -2.168839 -0.560547

6 -3.675119 -1.389788 -0.369451

6 -2.179222 -2.698803 -1.817784

6 -4.472712 -1.119270 -1.486581

6 -3.007765 -2.395058 -2.902922

6 -4.150679 -1.598975 -2.760859

1 -5.363550 -0.498957 -1.355292

1 -2.749836 -2.792829 -3.888474

6 0.243549 3.174935 -3.186795

1 0.055901 2.101831 -3.338234

1 -0.607215 3.729762 -3.605410

1 1.141201 3.434297 -3.772007

6 2.637516 2.372982 1.198443

1 2.295335 1.339674 1.370419

1 3.606002 2.319217 0.677316

1 2.791599 2.840907 2.179930

6 -0.946435 -3.541525 -2.000378

1 -0.043711 -2.909056 -1.934033

1 -0.855121 -4.302599 -1.210002

1 -0.956627 -4.046901 -2.976486

6 -4.037531 -0.849562 0.986528

1 -4.759739 -0.027979 0.901968

1 -4.488814 -1.628163 1.624325

1 -3.156771 -0.468733 1.518919

6 -1.138536 5.710295 0.976401

1 -0.575990 6.403345 1.621441

1 -1.726341 6.303710 0.260797

1 -1.844430 5.162169 1.623303

6 -4.993679 -1.237205 -3.956918

1 -4.932387 -2.004679 -4.743340

1 -6.051709 -1.107878 -3.682660

1 -4.652231 -0.285202 -4.399500

d-B

85

45 -0.688939 0.769053 0.392000

7 1.262193 0.599935 2.848002

7 0.168304 2.445084 2.703694

7 -0.194518 2.798652 -0.206851

7 -0.976904 1.212054 -2.523960

7 -2.602192 -0.083924 -1.972490

6 0.286210 1.219326 2.128710

6 1.748727 1.433089 3.849515

1 2.534843 1.115190 4.527445

6 1.062649 2.605493 3.754858

6 -0.679681 3.460910 2.094535

6 -0.194535 3.803548 0.705015

6 0.242004 5.084312 0.391134

1 0.221447 5.865395 1.152969

6 0.704396 5.351072 -0.905065

1 1.055804 6.348459 -1.176897

6 0.706461 4.318450 -1.833818

1 1.069982 4.473828 -2.850600

6 0.244215 3.048652 -1.466478

6 0.319052 1.902191 -2.433755

6 -1.530133 0.583058 -1.456784

6 -1.668810 0.937746 -3.691710

6 -2.702562 0.122030 -3.345452

1 1.121558 3.517166 4.342239

1 -0.682237 4.351683 2.734881

1 -1.701546 3.052131 2.029514

1 -1.363741 1.335467 -4.654845

1 -3.496203 -0.324542 -3.936256

6 -1.279573 -0.843521 1.019612

8 -1.634885 -1.849177 1.463190

6 1.406020 0.905242 -2.017348

6 2.392584 0.500814 -2.793557

6 -3.457567 -0.946042 -1.213505

6 -3.237980 -2.332223 -1.279243

6 -4.451293 -0.381640 -0.400617

6 -4.066493 -3.159780 -0.516890

6 -5.251407 -1.251380 0.349661

6 -5.075170 -2.639273 0.304453

1 -3.902825 -4.240430 -0.546592

1 -6.029665 -0.829838 0.991681

6 1.844408 -0.651101 2.443375

6 2.855327 -0.613432 1.467879

6 1.356767 -1.849541 2.981857

6 3.342993 -1.831928 0.986343

6 1.881715 -3.044133 2.475202

6 2.855895 -3.054584 1.468014

1 4.104226 -1.825261 0.204634

1 1.506446 -3.992308 2.870254

6 -2.100622 -2.892127 -2.092327

1 -2.227332 -2.700264 -3.169707

6 -4.609450 1.111482 -0.306475

1 -3.735301 1.548066 0.206712

6 3.397424 0.696937 0.962610

6 0.314553 -1.845757 4.069625

1 -0.541886 -1.207469 3.809817

1 1.191405 0.522558 -0.981743

6 3.324396 -0.555989 -2.566921

6 4.701941 -0.310867 -2.307298

6 2.922345 -1.920670 -2.639885

6 5.599652 -1.355999 -2.102404

1 5.049145 0.724863 -2.272384

6 3.833294 -2.957060 -2.458315

1 1.871794 -2.141943 -2.843908

6 5.181218 -2.692244 -2.178111

1 6.648313 -1.126817 -1.889787

1 3.485748 -3.992717 -2.521670

1 5.892899 -3.508746 -2.035583

1 3.860914 1.277330 1.777954

1 2.601885 1.318250 0.526140

1 -0.064362 -2.860625 4.252121

1 0.729038 -1.462714 5.017836

1 -2.012366 -3.977395 -1.947626

1 -1.145722 -2.425889 -1.799397

1 -4.659720 1.579093 -1.302259

1 -5.516870 1.379725 0.252383

1 4.145927 0.527245 0.179571

1 0.561431 2.278577 -3.432993

6 3.349104 -4.353169 0.884559

1 2.725137 -4.646547 0.023288

1 4.378143 -4.258957 0.509847

1 3.308957 -5.171118 1.620157

6 -5.918254 -3.556826 1.152686

1 -6.834893 -3.058873 1.501454

1 -6.207980 -4.464032 0.599996

1 -5.358161 -3.885538 2.044973

d-TSBC_BH

113

45 -1.645352 0.412368 0.959835

7 -3.291549 -2.234385 1.384629

7 -3.037241 -1.043440 3.154255

7 -0.405871 0.199924 2.745915

7 1.044337 1.609144 0.664134

7 -0.284736 2.955307 -0.348192

6 -2.802912 -1.039826 1.814541

6 -3.811603 -2.973066 2.442186

1 -4.247479 -3.956243 2.293826

6 -3.643745 -2.223554 3.565941

6 -2.491877 0.008827 4.000975

6 -0.979943 -0.000943 3.954115

6 -0.221831 -0.235143 5.097086

1 -0.719123 -0.390069 6.055965

6 1.172290 -0.271649 4.992317

1 1.791638 -0.455361 5.872752

6 1.755762 -0.069681 3.747950

1 2.837091 -0.095022 3.617314

6 0.940243 0.173784 2.634738

6 1.549021 0.367758 1.270604

6 -0.259859 1.785309 0.346855

6 1.837987 2.634362 0.179236

6 0.997256 3.494000 -0.459392

1 -3.911492 -2.417183 4.600581

1 -2.847994 -0.140450 5.027758

1 -2.862856 0.975625 3.626057

1 2.916777 2.621876 0.299628

1 1.185017 4.423486 -0.987295

6 -2.762328 0.660489 -0.465951

8 -3.489869 0.789418 -1.351470

6 1.282805 -0.811797 0.350052

6 2.244180 -1.337733 -0.413705

6 -1.453804 3.491472 -0.977690

6 -1.630382 3.263980 -2.353904

6 -2.398719 4.177690 -0.202639

6 -2.786502 3.769955 -2.952904

6 -3.543940 4.661151 -0.847884

6 -3.753631 4.469897 -2.218126

1 -2.947010 3.593918 -4.020086

1 -4.293499 5.197798 -0.260030

6 -3.122694 -2.725415 0.044083

6 -1.930692 -3.394995 -0.274393

6 -4.139972 -2.501565 -0.897353

6 -1.758389 -3.821986 -1.596347

6 -3.922001 -2.949302 -2.203193

6 -2.736524 -3.601633 -2.572283

1 -0.826954 -4.319540 -1.871072

1 -4.697031 -2.776343 -2.955063

6 -0.633206 2.446009 -3.131755

1 0.355011 2.929088 -3.182357

6 -2.202572 4.342025 1.280210

1 -2.297404 3.363939 1.782654

6 -0.877214 -3.675365 0.765016

6 -5.416389 -1.803929 -0.505821

1 -5.218543 -0.805434 -0.088641

1 0.216031 -1.105041 0.307098

6 1.948848 -2.320889 -1.449876

6 2.518755 -3.613738 -1.429871

6 1.129367 -1.985978 -2.552678

6 2.263969 -4.529993 -2.450639

1 3.168995 -3.887072 -0.594957

6 0.907496 -2.891532 -3.590331

1 0.677948 -0.992351 -2.585300

6 1.465124 -4.173281 -3.545033

1 2.708342 -5.528172 -2.403340

1 0.280789 -2.598530 -4.436718

1 1.289283 -4.883761 -4.356598

1 -1.166636 -4.534057 1.395551

1 -0.720068 -2.817198 1.432086

1 -6.076821 -1.680700 -1.374909

1 -5.966573 -2.371781 0.262775

1 -0.981414 2.275589 -4.159343

1 -0.473528 1.465657 -2.654448

1 -1.197573 4.724529 1.517184

1 -2.947764 5.028904 1.705086

1 0.083939 -3.906976 0.288017

1 2.629699 0.495783 1.360340

6 -2.515802 -4.033827 -3.999070

1 -3.403085 -4.544378 -4.406095

1 -2.319003 -3.160701 -4.643971

1 -1.652553 -4.707545 -4.085310

6 -5.001470 4.977383 -2.894762

1 -5.627440 4.139019 -3.243742

1 -5.610310 5.591310 -2.215410

1 -4.757078 5.586948 -3.779635

1 3.501550 -0.642164 -0.324626

14 4.826898 0.975841 -1.704921

14 5.434131 0.003314 1.182838

7 4.472231 0.182509 -0.221885

6 3.275378 1.030143 -2.790276

1 2.981663 0.013432 -3.094157

1 2.421944 1.476349 -2.257172

1 3.458401 1.618472 -3.705464

6 6.176378 0.109364 -2.718503

1 6.335377 0.597100 -3.695678

1 7.138511 0.103999 -2.181050

1 5.891919 -0.939963 -2.903348

6 5.422979 2.763580 -1.429497

1 6.333335 2.782246 -0.806837

1 5.664783 3.258856 -2.385478

1 4.663565 3.379664 -0.919268

6 4.913127 -1.578142 2.094957

1 3.818635 -1.660255 2.184196

1 5.245352 -2.461044 1.523923

1 5.356877 -1.639839 3.103149

6 7.298322 -0.113849 0.858413

1 7.677422 0.799778 0.371074

1 7.863797 -0.252856 1.795767

1 7.523214 -0.963468 0.193359

6 5.211412 1.474815 2.379853

1 5.790098 1.350145 3.311426

1 5.555141 2.404018 1.894389

1 4.156071 1.633417 2.660307

d-C_BH

113

45 1.136318 -0.615358 0.652155

7 3.350691 1.070101 2.147804

7 1.933210 0.098064 3.434273

7 -0.593608 -0.125777 1.882564

7 -1.438805 -0.973414 -0.777129

7 -0.214241 -2.642264 -1.337868

6 2.264207 0.249937 2.124191

6 3.684862 1.425582 3.450857

1 4.520260 2.083495 3.669639

6 2.784586 0.812183 4.266590

6 0.771142 -0.684934 3.835245

6 -0.505674 -0.145135 3.231328

6 -1.541952 0.313319 4.041551

1 -1.433505 0.283936 5.127136

6 -2.707585 0.800838 3.446237

1 -3.537496 1.160809 4.057990

6 -2.804553 0.791043 2.060553

1 -3.710619 1.086896 1.529732

6 -1.731199 0.308961 1.300968

6 -1.877963 0.291795 -0.200360

6 -0.202767 -1.493524 -0.610333

6 -2.231485 -1.764471 -1.597539

6 -1.455830 -2.824154 -1.954624

1 2.682495 0.815556 5.347926

1 0.705930 -0.674940 4.930011

1 0.921015 -1.724239 3.502907

1 -3.271162 -1.482467 -1.773029

1 -1.666003 -3.697325 -2.564088

6 2.562404 -1.019229 -0.414981

8 3.453297 -1.266707 -1.104606

6 -1.212207 1.446923 -0.880963

6 -1.141537 2.706519 -0.415412

6 0.875132 -3.568826 -1.406129

6 1.685663 -3.582960 -2.553431

6 1.110513 -4.416508 -0.312748

6 2.751784 -4.485711 -2.589303

6 2.192396 -5.302128 -0.394764

6 3.020053 -5.354000 -1.522274

1 3.400058 -4.500734 -3.469752

1 2.392436 -5.968728 0.448521

6 3.972064 1.605627 0.968874

6 3.372927 2.720359 0.357749

6 5.122977 0.991813 0.458007

6 3.952237 3.206813 -0.817042

6 5.668193 1.516345 -0.720524

6 5.095902 2.614872 -1.372382

1 3.487460 4.062039 -1.313690

1 6.555764 1.041393 -1.146339

6 1.437089 -2.616305 -3.680708

1 0.452042 -2.773854 -4.147721

6 0.249602 -4.342888 0.919783

1 0.452389 -3.404555 1.464123

6 2.137007 3.344712 0.943560

6 5.727235 -0.207810 1.138630

1 4.986630 -1.013961 1.254524

1 -0.802377 1.207500 -1.865840

6 -0.488397 3.835214 -1.086445

6 -0.691357 5.139562 -0.597716

6 0.389456 3.668814 -2.176156

6 -0.046297 6.235985 -1.171765

1 -1.366516 5.288188 0.249096

6 1.031699 4.762987 -2.751758

1 0.595602 2.667211 -2.557301

6 0.821264 6.054068 -2.252350

1 -0.222093 7.238312 -0.773380

1 1.711831 4.607137 -3.593120

1 1.330000 6.909645 -2.702498

1 2.254088 3.537267 2.021766

1 1.274175 2.670895 0.824284

1 6.571723 -0.603722 0.558553

1 6.093563 0.039989 2.148496

1 2.205279 -2.715208 -4.459690

1 1.451320 -1.577685 -3.313876

1 -0.822907 -4.333295 0.671910

1 0.447186 -5.189303 1.592035

1 1.894341 4.289322 0.442836

1 -3.019156 0.273699 -0.370507

6 5.692855 3.166512 -2.641968

1 6.139380 4.160466 -2.469921

1 6.479299 2.509372 -3.040238

1 4.923314 3.290843 -3.420486

6 4.169174 -6.326739 -1.601182

1 3.943817 -7.148636 -2.302081

1 5.085021 -5.834831 -1.965263

1 4.387832 -6.775738 -0.621412

1 -1.595103 2.937874 0.554001

14 -5.383683 -1.322441 0.551472

14 -5.376600 1.168857 -1.297087

7 -4.699854 -0.133196 -0.443158

6 -6.618923 -2.464770 -0.339543

1 -7.484807 -1.892136 -0.709943

1 -6.141771 -2.941029 -1.213085

1 -6.997522 -3.264684 0.320311

6 -6.320897 -0.626052 2.064709

1 -6.756556 -1.423501 2.691519

1 -5.658072 -0.021045 2.706876

1 -7.144441 0.030442 1.735750

6 -4.049492 -2.490267 1.266735

1 -3.286937 -1.949422 1.850074

1 -4.511461 -3.236239 1.935901

1 -3.527952 -3.038657 0.465265

6 -5.225200 2.807516 -0.326892

1 -5.746585 2.734226 0.643311

1 -4.165269 3.039339 -0.124199

1 -5.651004 3.665163 -0.875644

6 -4.437526 1.450551 -2.928314

1 -4.507790 0.561998 -3.578787

1 -4.831734 2.314627 -3.489902

1 -3.368328 1.642253 -2.735878

6 -7.216572 0.975302 -1.738047

1 -7.596822 1.848526 -2.295542

1 -7.377698 0.078470 -2.358968

1 -7.831101 0.862977 -0.828720

d-TSCD_BH

113

45 1.194337 -0.628011 0.680447

7 3.267482 1.209648 2.188748

7 1.949464 0.099380 3.470735

7 -0.562743 -0.236719 1.903481

7 -1.348461 -1.055407 -0.781283

7 -0.029595 -2.641070 -1.390238

6 2.263615 0.290795 2.162558

6 3.566652 1.588294 3.494151

1 4.336429 2.320653 3.717156

6 2.727975 0.888519 4.306824

6 0.827180 -0.751708 3.850182

6 -0.465312 -0.240163 3.253293

6 -1.483144 0.246634 4.067039

1 -1.366783 0.228573 5.151948

6 -2.639149 0.760516 3.471335

1 -3.454099 1.152225 4.083943

6 -2.747194 0.733348 2.089080

1 -3.650324 1.055021 1.572198

6 -1.700301 0.201889 1.316408

6 -1.879278 0.156169 -0.168147

6 -0.090992 -1.524530 -0.614280

6 -2.076524 -1.848831 -1.659535

6 -1.244014 -2.853062 -2.046120

1 2.626564 0.877068 5.388186

1 0.760171 -0.774577 4.944741

1 1.026336 -1.771012 3.485379

1 -3.119014 -1.616052 -1.867388

1 -1.397386 -3.703992 -2.702236

6 2.658117 -0.937755 -0.366256

8 3.583742 -1.123769 -1.029655

6 -1.400431 1.365230 -0.874068

6 -1.382751 2.634246 -0.414436

6 1.108930 -3.504102 -1.480294

6 1.932720 -3.426855 -2.615946

6 1.378816 -4.381528 -0.419005

6 3.046505 -4.268435 -2.674273

6 2.508536 -5.203142 -0.522258

6 3.350335 -5.163721 -1.639440

1 3.704954 -4.211440 -3.545466

1 2.735542 -5.891604 0.296259

6 3.807836 1.823808 1.007455

6 3.064703 2.851576 0.403132

6 5.020362 1.358210 0.480132

6 3.561186 3.403174 -0.782182

6 5.478938 1.944331 -0.705129

6 4.762712 2.960263 -1.351209

1 2.982566 4.188062 -1.275848

1 6.413259 1.583395 -1.143132

6 1.646811 -2.426939 -3.704814

1 0.684270 -2.624412 -4.202731

6 0.502722 -4.404794 0.804587

1 0.644209 -3.477560 1.386001

6 1.762853 3.314812 0.995079

6 5.775461 0.239977 1.148648

1 5.151696 -0.663353 1.233081

1 -1.053627 1.167151 -1.892772

6 -0.849381 3.796294 -1.128259

6 -1.097698 5.090451 -0.628993

6 -0.029250 3.683052 -2.270372

6 -0.556188 6.221630 -1.240840

1 -1.726976 5.201805 0.258136

6 0.509520 4.812265 -2.883599

1 0.214741 2.694862 -2.664405

6 0.253112 6.090713 -2.373044

1 -0.767365 7.212584 -0.830807

1 1.145984 4.694039 -3.764619

1 0.681348 6.973915 -2.852640

1 1.840305 3.461543 2.083614

1 0.975280 2.564773 0.820194

1 6.675476 -0.022410 0.576189

1 6.087875 0.512328 2.170005

1 2.436375 -2.441567 -4.468434

1 1.586435 -1.407575 -3.291608

1 -0.565773 -4.448090 0.543266

1 0.742255 -5.263519 1.446988

1 1.431674 4.253742 0.535491

1 -3.145140 -0.000060 -0.303233

6 5.280219 3.585240 -2.621738

1 5.838100 4.512941 -2.405652

1 5.961528 2.908175 -3.157953

1 4.455388 3.854013 -3.299083

6 4.557510 -6.061345 -1.739407

1 4.430018 -6.809771 -2.539708

1 5.464350 -5.483722 -1.980616

1 4.738262 -6.603307 -0.799855

1 -1.767925 2.841174 0.588991

14 -5.157419 -1.585614 0.585122

14 -5.370093 0.930069 -1.226895

7 -4.551507 -0.320056 -0.390228

6 -6.244266 -2.817658 -0.369420

1 -7.144865 -2.326581 -0.770872

1 -5.688486 -3.239840 -1.223848

1 -6.571635 -3.655406 0.270129

6 -6.195278 -0.969940 2.059646

1 -6.574609 -1.801336 2.678348

1 -5.600836 -0.308953 2.712842

1 -7.064482 -0.389281 1.707847

6 -3.742446 -2.623556 1.329870

1 -3.103060 -2.039956 2.010064

1 -4.163984 -3.461407 1.910753

1 -3.097943 -3.051653 0.545484

6 -5.347039 2.562411 -0.246345

1 -5.833753 2.434575 0.735895

1 -4.309125 2.892796 -0.073210

1 -5.868581 3.376067 -0.778603

6 -4.514111 1.285461 -2.882997

1 -4.516205 0.389825 -3.527132

1 -5.013787 2.100777 -3.433020

1 -3.466082 1.586932 -2.724348

6 -7.189958 0.538413 -1.600478

1 -7.675724 1.378941 -2.124711

1 -7.278532 -0.355667 -2.238971

1 -7.759427 0.346067 -0.675996

D_BH

113

45 1.941681 -0.044371 0.638325

7 3.387187 2.609124 1.489825

7 2.325079 1.625783 3.076510

7 0.067268 0.230493 1.703314

7 -0.368855 -1.456056 -0.610853

7 1.358321 -2.744904 -0.796606

6 2.672209 1.485073 1.773211

6 3.467438 3.443443 2.602583

1 3.990661 4.394126 2.571847

6 2.786867 2.822286 3.605184

6 1.419808 0.663859 3.698118

6 0.083871 0.665525 2.991608

6 -1.028127 1.163625 3.642841

1 -0.953052 1.506636 4.675421

6 -2.252125 1.206635 2.937585

1 -3.163529 1.559183 3.425808

6 -2.295041 0.749636 1.644809

1 -3.242406 0.678888 1.118210

6 -1.127630 0.218991 1.010464

6 -1.241167 -0.363304 -0.285649

6 0.964211 -1.509753 -0.359682

6 -0.806744 -2.645204 -1.193162

6 0.281929 -3.450568 -1.318047

1 2.600397 3.121263 4.632547

1 1.296781 0.932967 4.754196

1 1.884751 -0.331577 3.631802

1 -1.849062 -2.820851 -1.433298

1 0.387441 -4.458840 -1.706239

6 3.515868 -0.243621 -0.264765

8 4.526180 -0.343339 -0.816868

6 -2.155209 0.022368 -1.305383

6 -2.979933 1.116427 -1.394448

6 2.697948 -3.242950 -0.718347

6 3.495333 -3.225592 -1.875171

6 3.181860 -3.707099 0.514356

6 4.804786 -3.702541 -1.775612

6 4.502516 -4.169966 0.565866

6 5.327262 -4.176135 -0.564696

1 5.441309 -3.684645 -2.664598

1 4.896308 -4.533066 1.519100

6 3.897904 2.924448 0.186344

6 2.996760 3.346793 -0.807646

6 5.268672 2.765768 -0.061692

6 3.516205 3.626902 -2.074968

6 5.739918 3.062702 -1.345979

6 4.881703 3.493837 -2.363057

1 2.831012 3.953095 -2.862360

1 6.804329 2.934901 -1.559269

6 2.965228 -2.652537 -3.162656

1 2.111365 -3.229996 -3.551430

6 2.317775 -3.664642 1.744951

1 2.152632 -2.616400 2.049110

6 1.520384 3.444653 -0.531982

6 6.189750 2.231977 1.003375

1 5.858903 1.234768 1.335582

1 -2.146129 -0.640380 -2.176321

6 -3.905074 1.398381 -2.480327

6 -4.543711 2.660951 -2.534811

6 -4.240196 0.469001 -3.494853

6 -5.455657 2.977859 -3.539637

1 -4.302914 3.402687 -1.768812

6 -5.147735 0.790056 -4.500977

1 -3.796046 -0.527724 -3.485363

6 -5.767328 2.045272 -4.534506

1 -5.927699 3.964118 -3.547432

1 -5.384264 0.045503 -5.266093

1 -6.484884 2.289517 -5.321037

1 1.313966 3.946492 0.425830

1 1.081036 2.434078 -0.462772

1 7.216450 2.140601 0.623474

1 6.212084 2.876998 1.896118

1 3.745865 -2.634651 -3.935426

1 2.606893 -1.621983 -3.009534

1 1.322916 -4.098292 1.559240

1 2.787056 -4.205599 2.578469

1 1.003801 3.991342 -1.332549

1 -4.890883 0.056272 -0.450368

6 5.400261 3.800934 -3.744864

1 4.903934 3.172529 -4.502396

1 5.203945 4.850629 -4.019383

1 6.483631 3.628485 -3.818790

6 6.755939 -4.650956 -0.485145

1 6.964211 -5.151200 0.471819

1 6.990773 -5.356064 -1.298431

1 7.456672 -3.804055 -0.580005

1 -2.937527 1.880470 -0.615347

14 -5.138003 -2.153301 0.316003

14 -6.384280 0.608921 1.294002

7 -5.417184 -0.429836 0.277037

6 -4.767455 -2.736418 -1.439766

1 -5.625398 -2.552219 -2.105408

1 -3.908816 -2.188289 -1.855049

1 -4.533807 -3.813709 -1.463220

6 -6.689435 -3.027059 0.942558

1 -6.536177 -4.118799 0.961607

1 -6.955120 -2.714075 1.964729

1 -7.550926 -2.814699 0.289356

6 -3.691105 -2.609056 1.438678

1 -3.883037 -2.277581 2.472033

1 -3.516251 -3.698018 1.455864

1 -2.767511 -2.115310 1.099050

6 -5.811104 2.384244 1.028413

1 -4.761098 2.526181 1.330560

1 -5.896219 2.660373 -0.034678

1 -6.430150 3.085429 1.611823

6 -8.218716 0.486470 0.869916

1 -8.587535 -0.542259 1.011038

1 -8.828320 1.154714 1.501232

1 -8.387861 0.761552 -0.184065

6 -6.151092 0.117711 3.104470

1 -6.703969 0.795967 3.775460

1 -6.515258 -0.904431 3.295278

1 -5.086011 0.146912 3.383713
